# Estimation of COVID-19 vaccine effectiveness against hospitalisation in individuals aged ≥ 65 years using electronic health registries; a pilot study in four EU/EEA countries, October 2021 to March 2022

**DOI:** 10.2807/1560-7917.ES.2022.27.30.2200551

**Published:** 2022-07-28

**Authors:** Alexis Sentís, Irina Kislaya, Nathalie Nicolay, Hinta Meijerink, Jostein Starrfelt, Iván Martínez-Baz, Jesús Castilla, Katrine Finderup Nielsen, Christian Holm Hansen, Hanne-Dorthe Emborg, Anthony Nardone, Tarik Derrough, Marta Valenciano, Baltazar Nunes, Susana Monge, the VEBIS-Lot4 working group, VEBIS-Lot4 working group, Ausenda Machado, Carlos Dias, Itziar Casado, Cristina Burgui, Amparo Larrauri, Clara Mazagatos

**Affiliations:** 1 Epidemiology Department, Epiconcept, Paris, France; 2 Instituto Nacional de Saúde Doutor Ricardo Jorge (INSA), Lisbon, Portugal; 3 Vaccine preventable diseases and Immunisation, European Centre for Disease Prevention and Control (ECDC), Solna, Sweden; 4 Norwegian Institute of Public Health (NIPH), Oslo, Norway; 5 Instituto de Salud Pública de Navarra - IdiSNA, Pamplona, Spain; 6 Statens Serum Institut (SSI), Copenhagen, Denmark; 7 Instituto de Salud Carlos III (ISCIII), Madrid, Spain; 8 CIBER on Epidemiology and Public Health (CIBERESP), Madrid, Spain; 9 CIBER on Infectious Diseases (CIBERINFEC), Madrid, Spain; 10 Members of the VEBIS-Lot4 working group are listed under Collaborators

**Keywords:** vaccine effectiveness, delta variant, omicron variant, vaccination primary course, vaccination booster dose, COVID-19

## Abstract

By employing a common protocol and data from electronic health registries in Denmark, Navarre (Spain), Norway and Portugal, we estimated vaccine effectiveness (VE) against hospitalisation due to COVID-19 in individuals aged ≥ 65 years old, without previous documented infection, between October 2021 and March 2022. VE was higher in 65–79-year-olds compared with ≥ 80-year-olds and in those who received a booster compared with those who were primary vaccinated. VE remained high (ca 80%) between ≥ 12 and < 24 weeks after the first booster administration, and after Omicron became dominant.

Using data from electronic health registries (EHR) in Denmark, Navarre (Spain), Norway and Portugal, we performed a pilot study to investigate vaccine effectiveness (VE) in the community against hospitalisation due to coronavirus disease (COVID-19) in people aged ≥ 65 years old without previous documented infection. The study period was between October 2021 and March 2022, coinciding with the rollout of the first booster dose in people ≥ 65 years old (October–December 2021) [1], and with the emergence of Omicron (Phylogenetic Assignment of Named Global Outbreak (Pango) lineage: B.1.1.529) severe acute respiratory syndrome coronavirus 2 (SARS-CoV-2) variant (December 2021–January 2022) [2]. Monthly effectiveness estimates of complete primary vaccination and of the first booster vaccination are presented.

## Development of a common protocol to estimate vaccine effectiveness using electronic health registries

Using a common protocol (Supplement S1. Study protocol), site-specific VE estimates were calculated in Denmark, Navarre (Spain), Norway and Portugal. We constructed cohort studies using individual deterministic linkage of EHR and administrative databases of resident population, vaccination status, SARS-CoV-2 tests, hospitalisations and clinical data. Individuals with a previous positive SARS-CoV-2 test at cohort enrolment and those living in long-term-care facilities were excluded, as were those vaccinated before the bulk of their age group (except in Denmark).

VE against hospitalisation due to COVID-19 was estimated in two different age groups respectively comprising 65 to 79-year-olds and in ≥ 80-year-olds. Hospitalisation due to COVID-19 was defined as laboratory-confirmed infection with SARS-CoV-2 and admission to hospital 24 hours before (48 hours in Denmark) or up to 3 weeks after the positive test or symptoms onset (2 weeks in Denmark), in which admission or discharge criteria were compatible with severe acute respiratory infection (SARI; based on similar criteria as in SARI surveillance, International Classification of Diseases (ICD) coding or similar recognised coding). We defined the date of the event (i.e. hospitalisation) as the date of the first SARS-CoV-2 positive test for that hospitalisation and not the date of hospitalisation.

The reference group comprised non-vaccinated individuals. Primary vaccination status was assigned to individuals after ≥ 7 or ≥ 14 days of one dose of COVID-19 Vaccine Janssen (Ad26.COV2.S, Janssen-Cilag International NV, Beerse, Belgium), or two doses of Vaxzevria (ChAdOx1 nCoV-19, AstraZeneca, Cambridge, United Kingdom), Comirnaty (BNT162b2, BioNTech-Pfizer, Mainz, Germany/New York, United States) or Spikevax (mRNA-1273, Moderna, Cambridge, United States) vaccines, administered 19 days apart. Booster vaccination status was achieved ≥ 7 days after an additional dose of Comirnaty or Spikevax, administered ≥ 3 months after primary vaccination.

Between October 2021 and March 2022, we reported five monthly VE estimates using an 8-week-wide observation window which was moved 1 month forward for each successive estimate. We used a survival analysis framework with time-zero (*t_0_*) on the first calendar day of each 8-week window. Follow-up ended upon death, discontinuation of registration, a positive SARS-CoV-2 test or administrative censoring (8 weeks after *t_0_*). Positive tests that led to hospitalisation were recorded as events. We used Cox proportional hazards regression model (calendar time scale) to estimate hazard ratios (HR) of hospitalisation adjusted by age (5-year age groups), sex, region, socioeconomic conditions (except for Denmark and Navarre) and comorbidities. The last two were defined differently according to data availability and relevance in each site (Supplement S1. Study protocol). HR was estimated in both age groups (65‒79 and ≥ 80 years) and time since first booster (< 12 weeks or ≥ 12– < 24 weeks). VE was (1 − HR) × 100. We pooled VE from the four sites using the generic inverse variance weighting meta-analysis method, using sites as random effects [3].

## Vaccine effectiveness against hospitalisation due to COVID-19 in individuals ≥ 65 years old without previous documented infection

The number of 65–79-year-olds included decreased from 3.1 million (October–November 2021) to 180,000 (February–March 2022) in the primary vaccinated group and increased from 280,000 to 2.8 million in the booster group. The corresponding number of events varied from 769 to 334, and from 48 to 1,972 respectively. Similarly, the number of individuals ≥ 80 years old decreased from 1.1 million (October–November 2021) to 76,000 (February–March 2022) in the primary vaccinated group and increased from 600,000 to 1.1 million in the booster group. The number of events varied from 652 to 399, and from 38 to 2,493 respectively (Supplement S2. Events and person-months).

**Figure 1:**
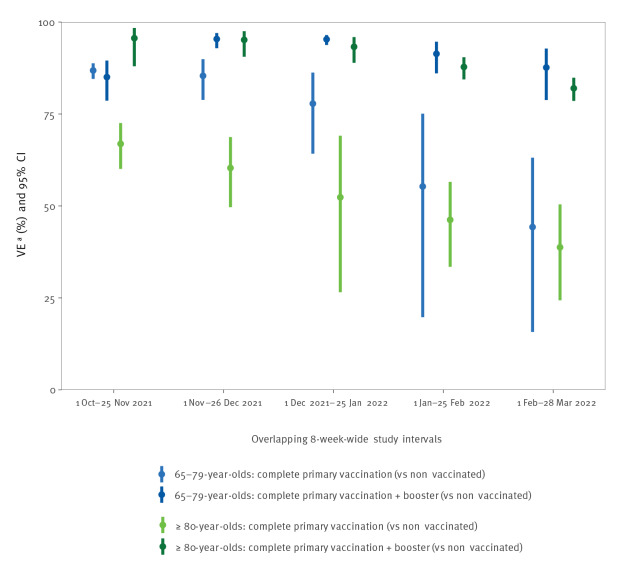
Vaccine effectiveness against hospitalisation due to COVID-19 in 65–79 and ≥ 80-year-olds, in overlapping 8-week-wide observation intervals, based on pooled estimates from Denmark, Navarre (Spain), Portugal and Norway, 1 October 2021‒28 March 2022^a^

CI: confidence interval; COVID-19: coronavirus disease; VE: vaccine effectiveness.

^a^ The values of VE were obtained by pooling VE estimates from Denmark, Portugal, Navarre (Spain) and Norway using random-effects meta-analysis. The supplementary material includes numbers supporting VE calculations (Supplement S2. Events and person-months), as well as the sample size per period and site (Supplement S3. Detailed site estimates).

In October–November 2021, primary vaccination VE was lower in ≥ 80-year-olds (67%; 95% confidence interval (CI): 60–73%) compared with 65–79-year-olds (87%; 95% CI: 85–89%). VE decreased over time and reached similar level in both age groups by February–March 2022,  namely 39% (95% CI: 24–50%) in ≥ 80-year-olds and 44% (95% CI: 16–63%) in 65–79-year-olds (Figure 1, Table 1).

In the graph, each VE figures as a dot, which is overlapped by a vertical line representing the respective 95% CI.

**Table 1:** Vaccine effectiveness against hospitalisation due to COVID-19 in 65–79 and ≥ 80-year-olds, in overlapping 8-week-wide observation intervals, based on pooled estimates from Denmark, Navarre (Spain), Portugal and Norway, 1 October 2021–28 March 2022^a^

Age in years	Observation period	VE of complete primary vaccination^a^		VE of complete primary vaccination + first booster^a^	
		Events/person-months^b^	VE (95% CI)	Events/person-months^c^	VE (95% CI)
**65–79**	1 Oct–25 Nov 2021	769/5,430,697	86.8% (84.5–88.8)	48/132,388	85.0% (78.6–89.5)
	1 Nov–26 Dec 2021	1,141/3,627,044	85.4% (78.8–89.9)	169/1,199,370	95.4% (92.9–97.0)
	1 Dec 2021–25 Jan 2022	766/1,261,165	77.8% (64.2–86.3)	597/3,623,380	95.3% (93.8–96.5)
	1 Jan–25 Feb 2022	515/386,221	55.3% (19.7–75.1)	1,429/4,981,833	91.4% (86.0–94.7)
	1 Feb 1–28 Mar 2022	334/261,264	44.3% (15.7–63.1)	1,972/4,783,200	87.6% (78.8–92.8)
**≥ 80**	1 Oct–25 Nov 2021	652/1,561,711	66.9% (60.1–72.6)	38/308,135	95.6% (88.0–98.4)
	1 Nov–26 Dec 2021	751/716,577	60.3% (49.7–68.7)	181/1,124,695	95.2% (90.6–97.5)
	1 Dec 2021–25 Jan 2022	620/262,138	52.4% (26.5–69.1)	884/1,752,544	93.3% (88.9–95.9)
	1 Jan–25 Feb 2022	544/148,922	46.2% (33.4–56.6)	1,938/1,901,312	87.8% (84.4–90.4)
	1 Feb–28 Mar 2022	399/117,721	38.8% (24.3–50.4)	2,493/1,819,768	82.0% (78.6–84.9)

CI: confidence interval; COVID-19: coronavirus disease; VE: vaccine effectiveness.

^a^ The values of VE were obtained by pooling VE estimates from Denmark, Portugal, Navarre (Spain) and Norway using random-effects meta-analysis. The supplementary material includes supporting numbers for the VE calculations (Supplement S2. Events and person-months), as well as the sample size per period and site (Supplement S3. Detailed site estimates).

^b^ The events per person-months presented in the column concern people who had primary vaccination status.

^c^ The events per person-months presented in the column concern people who, following complete primary vaccination, had also received the first booster and acquired booster vaccination status.

**Figure 2:**
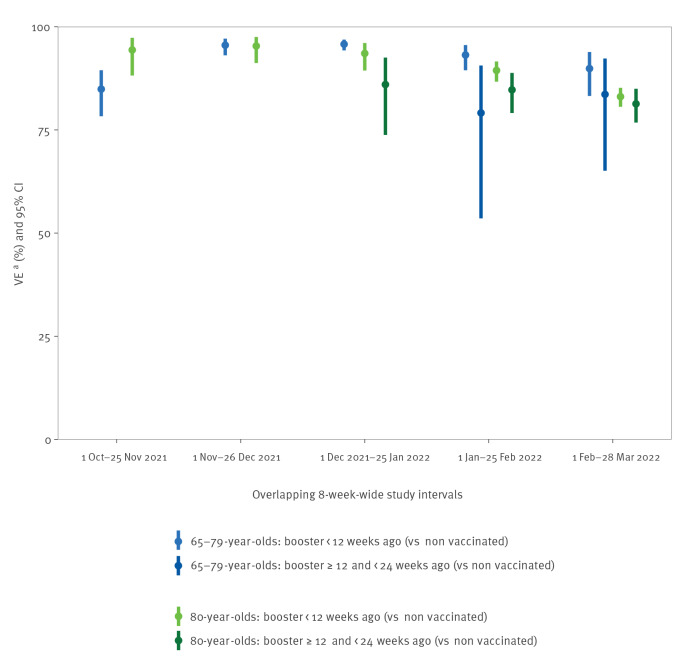
Vaccine effectiveness against hospitalisation due to COVID-19, by time since acquiring first booster vaccination status, in 65–79 and ≥ 80-year-olds, in overlapping 8-week-wide observation intervals, based on pooled estimates from Denmark, Navarre (Spain), Portugal and Norway, 1 October 2021‒28 March 2022^a^

CI: confidence interval; COVID-19: coronavirus disease; VE: vaccine effectiveness.

^a^ The values of VE were obtained by pooling VE estimates from Denmark, Navarre (Spain), Norway and Portugal, using random-effects meta-analysis. The number of events and person-month per period are described in Table 2 and the sample size per period and site can be found in the supplemental material (Supplement S3. Detailed site estimates).

Highest VE for the booster dose was achieved when the bulk of the age group had been vaccinated, in October–November 2021 for the ≥ 80-year-olds (VE: 96%; 95% CI: 88–98%), and in November–December 2021 for the 65–79-year-olds (VE: 95%; 95% CI: 93–97%). VE declined thereafter, although it remained high, at 82% (95% CI: 79–85%) in ≥ 80-year-olds and 88% (95% CI: 79–93%) in the 65–79-year-olds, in February–March 2022 (Figure 1, Table 1). By time since acquiring booster vaccination status, VE appeared to be generally higher within the first 12 weeks than between 12 and 24 weeks, though it remained high, at around 80% in February–March 2022. For VE within 12 weeks post-boosting, the estimations appeared to slightly decrease over time during the study period (Figure 2, Table 2).

In the graph, each VE figures as a dot, which is overlapped by a vertical line representing the respective 95% CI. For the first two observation intervals (October‒November and November‒December 2021), there were too few data to estimate VE ≥ 12 weeks post-acquiring booster vaccination status in individuals aged ≥ 65 years old. This remained the case for the 65‒79-year-old age group during the third observation interval (December 2021‒January 2022), but not for the ≥ 80-year-old age group.

**Table 2:** Estimated vaccine effectiveness against hospitalisation due to COVID-19, by time since acquiring first booster vaccination status, in 65–79 and ≥ 80-year-olds, in overlapping 8-week-wide observation intervals, 1 October 2021‒28 March 2022^a^

Age in years	Observation period	VE of complete primary vaccination + first booster < 12 weeks ago^a^		VE of complete primary vaccination + first booster ≥ 12 and < 24 weeks ago^a^	
		Events/person-months^b^	VE (95% CI)	Events/person-months^c^	VE (95% CI)
**65–79**	1 Oct‒25 Nov 2021	47/132,020	84.9% (78.3‒89.5)	1/351	NA
	1 Nov‒26 Dec 2021	161/1,196,736	95.5% (93.1‒97.1)	8/2,579	NA
	1 Dec 2021‒25 Jan 2022	518/3,594,550	95.8% (94.3‒96.9)	79/28,640	NA
	1 Jan‒25 Feb 2022	997/4,424,909	93.2% (89.5‒95.6)	432/256,862	79.1% (53.6‒90.6)
	1 Feb‒28 Mar 2022	941/3,226,817	89.9% (83.2‒93.9)	1,028/1,674,165	83.6% (65.1‒92.3)
**≥ 80**	1 Oct‒25 Nov 2021	37/308,023	94.4% (88.2‒97.3)	1/89	NA
	1 Nov‒26 Dec 2021	180/1,124,099	95.3% (91.2‒97.5)	1/545	NA
	1 Dec 2021‒25 Jan 2022	824/1,723,446	93.5% (89.4‒96.1)	60/29,017	86.0% (73.8‒92.5)
	1 Jan‒25 Feb 2022	1,136/1,398,267	89.4% (86.7‒91.6)	802/503,004	84.7% (79.1‒88.8)
	1 Feb‒28 Mar 2022	657/599,118	83.1% (80.6‒85.2)	1,877/1,271,389	81.3% (76.8‒85.0)

COVID-19: coronavirus disease; CI: confidence interval; NA: not available; VE: vaccine effectiveness.

^a^ The values of VE were obtained by pooling VE estimates from Denmark, Navarre (Spain), Norway and Portugal using random-effects meta-analysis. The sample size per period and site can be found in the supplemental material (Supplement S3. Detailed site estimates).

^b^ The events per person-months presented in the column concern people who, following complete primary vaccination, further acquired booster vaccination status < 12 weeks ago.

^c^ The events per person-months presented in the column concern people who, following complete primary vaccination, further acquired booster vaccination status ≥ 12 and < 24 weeks ago.

We used I^2^ to measure the proportion of total variance attributable to the random site variability vs within site variability. I^2^ values were > 41% (except when all site-specific CIs overlapped and I^2^ = 0%) and frequently they were > 70%, indicating a considerable heterogeneity between sites (Supplement S3. Detailed site estimates). Using fixed-effects meta-analysis the point estimates did not significantly change, but estimated CIs were narrower (Supplement S4. Fixed effects meta-analysis).

## Discussion

In late 2020, the European Centre for Disease Prevention and Control (ECDC) established the Vaccine Effectiveness, Burden and Impact Studies of COVID-19 and Influenza (VEBIS) programme to monitor COVID-19 VE and detect VE variations that required further investigation and inform public health action [4]. One component of VEBIS is based on estimating COVID-19 VE using routinely collected exposure and outcome data from EHR.

Accordingly, this pilot study used EHR data originating from four European Union/European Economic Area (EA/EEA) countries, to investigate VE against hospitalisation due to COVID-19 in people from the community aged ≥ 65 years without previous documented infection. Our results indicate that VE of primary vaccination decreases over time. This is likely due to a combination of waning of immunity, but the emergence of Omicron variants during the study period could have also played a role [2,5]. Lower VE in ≥ 80-year-olds compared to the 65‒79-year-olds in the first months of study period may be explained by waning of immunity in this earlier vaccinated group [5,6] and/or a weaker immunological response to vaccines with increasing age [7,8]. Nonetheless, different vaccine brands and schedules may have contributed to the observed differences in the VE estimates between the two age groups and this will be the subject of further investigation in the coming months when more data will be available.

Administration of a booster dose restored VE to levels comparable to those reported after primary vaccination [9,10]. In both age groups, the higher VE in the booster group compared with primary vaccination was evident both before and after the emergence of Omicron, supporting the policy of booster administration to limit the impact of the Omicron-dominated epidemic [10-12]. Over the study period, the effect of the booster appeared to decrease with time, even for those boosted since less than 12 weeks. This may reflect the emergence of Omicron variant. However, it may also represent waning protection soon after the booster dose, as reported by other studies [13,14]. In this respect, VE estimates in our study seemed lower beyond 12 weeks of acquiring booster vaccination status, even though protection remained high (ca 80%) and the distribution of time since the booster was skewed toward the 12-week limit.

There was high heterogeneity in the pooled estimates, despite the use of a common protocol. Vaccination rollout was similar in all sites but there were some differences in vaccine brands and type of vaccination schedules used, as well as in local SARS-CoV-2 epidemiology, especially with the emergence of Omicron sub-variants BA.1 and BA.2 [2]. Moreover, we did not account for changing testing recommendations or control measures in the period. These and other unmeasured factors could result in a variable VE across sites. Nevertheless, we demonstrated how the use of a common methodology applied to national EHR can provide rapid and almost real-time VE estimates for public health workers and policymakers to monitor the impact of the vaccination strategy at the European level.

## Conclusion

The results of this VE pilot study bring additional information to the public health benefit of the first booster dose to prevent severe outcome of COVID-19 in individuals aged ≥ 65 years old and serve as a proof of concept for a new infrastructure for COVID-19 VE monitoring in Europe using EHRs.

## Supplementary Data

2045000 Supplementary Material

## References

[1] European Centre for Disease Prevention and Control (ECDC). Overview of the implementation of COVID-19 vaccination strategies and deployment plans in the EU/EEA. Stockholm: ECDC; 21 Apr 2022. [Accessed 13 Jun 2022]. Available from: https://www.ecdc.europa.eu/en/publications-data/overview-implementation-covid-19-vaccination-strategies-and-deployment-plans

[2] European Centre for Disease Prevention and Control (ECDC). Weekly epidemiological update: Omicron variant of concern (VOC) – week 2 (data as of 20 January 2022) EU/EEA. Stockholm: ECDC; 21 Jan 2022. [Accessed 16 Jun 2022]. Available from: https://www.ecdc.europa.eu/en/news-events/weekly-epidemiological-update-omicron-variant-concern-voc-week-2-data-20-january-2022

[3] Chapter 10: Analysing data and undertaking meta-analyses. Cochrane Training. [Accessed 13 Jun 2022]. Available from: https://training.cochrane.org/handbook/archive/v6.2/chapter-10#section-10-3

[4] European Centre for Disease Prevention and Control (ECDC). Engagement of ECDC in projects funded through the Horizon 2020 call for Pan-European COVID-19 cohorts. Stockholm: ECDC; 9 Jun 2020. Available from: https://www.ecdc.europa.eu/en/news-events/engagement-ECDC-projects-funded-through-call-for-Pan-European-COVID-19-cohorts

[5] Goldberg Y Mandel M Bar-On YM Bodenheimer O Freedman L Haas EJ Waning Immunity after the BNT162b2 Vaccine in Israel. N Engl J Med. 2021;385(24):e85. 10.1056/NEJMoa2114228 34706170 PMC8609604

[6] Fabiani M Puopolo M Morciano C Spuri M Spila Alegiani S Filia A Effectiveness of mRNA vaccines and waning of protection against SARS-CoV-2 infection and severe covid-19 during predominant circulation of the delta variant in Italy: retrospective cohort study. BMJ. 2022;376:e069052. 10.1136/bmj-2021-069052 35144968 PMC8829820

[7] Nanishi E Levy O Ozonoff A . Waning effectiveness of SARS-CoV-2 mRNA vaccines in older adults: a rapid review. Hum Vaccin Immunother. 2022;18(5):2045857. 10.1080/21645515.2022.2045857 35240940 PMC9196671

[8] Andrews N Tessier E Stowe J Gower C Kirsebom F Simmons R Duration of Protection against Mild and Severe Disease by Covid-19 Vaccines. N Engl J Med. 2022;386(4):340-50. 10.1056/NEJMoa2115481 35021002 PMC8781262

[9] Andrews N Stowe J Kirsebom F Toffa S Rickeard T Gallagher E Covid-19 Vaccine Effectiveness against the Omicron (B.1.1.529) Variant. N Engl J Med. 2022;386(16):1532-46. 10.1056/NEJMoa2119451 35249272 PMC8908811

[10] Barda N Dagan N Cohen C Hernán MA Lipsitch M Kohane IS Effectiveness of a third dose of the BNT162b2 mRNA COVID-19 vaccine for preventing severe outcomes in Israel: an observational study. Lancet. 2021;398(10316):2093-100. 10.1016/S0140-6736(21)02249-2 34756184 PMC8555967

[11] Arbel R Hammerman A Sergienko R Friger M Peretz A Netzer D BNT162b2 Vaccine Booster and Mortality Due to Covid-19. N Engl J Med. 2021;385(26):2413-20. 10.1056/NEJMoa2115624 34879190 PMC8728797

[12] Abu-Raddad LJ Chemaitelly H Ayoub HH AlMukdad S Yassine HM Al-Khatib HA Effect of mRNA Vaccine Boosters against SARS-CoV-2 Omicron Infection in Qatar. N Engl J Med. 2022;386(19):1804-16. 10.1056/NEJMoa2200797 35263534 PMC8929389

[13] Ferdinands JM Rao S Dixon BE Mitchell PK DeSilva MB Irving SA Waning 2-Dose and 3-Dose Effectiveness of mRNA Vaccines Against COVID-19-Associated Emergency Department and Urgent Care Encounters and Hospitalizations Among Adults During Periods of Delta and Omicron Variant Predominance - VISION Network, 10 States, August 2021-January 2022. MMWR Morb Mortal Wkly Rep. 2022;71(7):255-63. 10.15585/mmwr.mm7107e2 35176007 PMC8853475

[14] Patalon T Saciuk Y Peretz A Perez G Lurie Y Maor Y Waning effectiveness of the third dose of the BNT162b2 mRNA COVID-19 vaccine. Nat Commun. 2022;13(1):3203. 10.1038/s41467-022-30884-6 35680872 PMC9184525

