# Supplementary material for: Estimation of COVID-19 vaccine effectiveness against hospitalisation in individuals aged ≥ 65 years using electronic health registries; a pilot study in four EU/EEA countries, October 2021 to March 2022
Source: Euro Surveill. 2022 Jul 28;27(30):2200551. doi: 10.2807/1560-7917.ES.2022.27.30.2200551 (PMC9336167; doi:10.2807/1560-7917.ES.2022.27.30.2200551)
Supplement: Supplementary Material [file 2200551_SupplementaryMaterial.pdf]

This supplementary material is hosted by *Eurosurveillance* as supporting information alongside the article 'Estimation of COVID-19 vaccine effectiveness against hospitalisation in individuals aged ≥ 65 years using electronic health registries; a pilot study in four EU/EEA countries, October 2021 to March 2022' on behalf of the authors who remain responsible for the accuracy and appropriateness of the content. The same standards for ethics, copyright, attributions and permissions as for the article apply. Supplements are not edited by Eurosurveillance and the journal is not responsible for the maintenance of any links or email addresses provided therein.

SUPPLEMENTARY MATERIAL

COVID-19 vaccine effectiveness against hospitalisation in individuals aged 65+ years using electronic health registries; pilot study in four EU/EEA countries, October-March 2022

Table of contents

Supplement S1. Summary of the study protocol 2

Supplement S2. Number of events and person-months of follow-up in the fixed-effects model, in each of the analyses, by site. 9

Supplement S3. Detailed site estimates and pooled results using either fixed or random effects meta-analysis. 11

Supplement S4. Results of the sensitivity analysis using fixed-effects meta-analysis instead of random-effects. Estimated vaccine effectiveness against hospitalisation due to COVID-19 (and 95% confidence intervals), in November 2021 - March 2022, in Denmark, Portugal, Navarre and Norway. 21

Supplement S1. Summary of the study protocol

1. Study period and setting

The study covers 8-week rolling study periods for VE monitoring over time according to the following table:

Table 1. Rolling study periods

Monthly estimate (data extraction)	Study/Follow-up period
January 2022	October 1 to November 28, 2021
February 2022	November 1 to December 26, 2021
March 2022	December 1, 2021 to January 25, 2022
April 2022	January 1 to February 25, 2022
May 2022	February 1 to March 28, 2022
June 2022	March 1 to April 25, 2022

Four study sites participate in the study: Denmark, Navarre (Spain), Norway and Portugal, all with population-based registries covering the entire population in their territories. This is a data-linkage study that involves different databases in each of the participating sites, all of which can be deterministically linked using individual identifiers.

In **Portugal**, information sources used for this study cover approximately 7.5 million inhabitants, 2.4 million over 65 years of age. Information on hospitalization is extracted from the BI-SINAVE +BIMH databases, while BIMH episodes are coded at discharge, so there is some delay in coding, and only public hospitals are covered. Date of laboratory confirmation of SARS-CoV-2 is obtained from BI-SINAVE and date and cause of hospital admission are obtained from BIMH. Information on vaccination is extracted from the VACINAS, a nationwide EHR with data on COVID-19 and other vaccines administered in Portugal, containing information on brand and date of uptake of each dose of COVID-19 vaccine and other vaccines. Information on covariates is obtained from the NHSU database, which is used as the central source system, therefore all users included in the study come from this source. Data on sex, date of birth, address and date of death are obtained from NHSU. The number of chronic conditions is extracted from Primary Care Information System (SIM@SNS) database, that allows to identify comorbidities including Anaemia, Dementia, Diabetes, Cardiac disease, neuromuscular disease, rheumatologic disease, obesity, tuberculosis, stroke, pulmonary disease except asthma, liver disease and hypertension belong to the group without immunosuppression, and HIV, Renal disease and Cancer belong to the group with immunosuppression. Data on the number of comorbidities is available, but due to data protection issues data on each individual comorbidity is not accessible. European deprivation index for Portugal at municipality level (Ribeiro AI et al 2018) is obtained from the Census 2011, but will be updated when the Census 2021 will be available.

In **Denmark**, information sources used for this study cover approximately 5 million inhabitants, around 1.18 million over 65 years of age. Information on hospitalizations will be extracted from the Danish National Patient Register (DNPR) and cross-matched with the Danish Microbiology Database (MiBA). In LPR the cause of hospitalization as COVID-19 or not COVID-19 related can be coded based on COVID-19 specific ICD-10 codes. Vaccination data will be extracted from the Danish Vaccination Registry (DDV), where information on uptake of other vaccines is also available. The LPR also has information on comorbidities to be used for adjustment and the Danish Civil Registration System (CPR) provides demographic information.

In **Navarre**, information sources used for this study cover approximately 650.000 inhabitants, around 123.000 over 65 years of age. Information on hospitalization is extracted from the Hospital admissions database, where a Public Health medical doctor reviews all hospitalized patients with a COVID-19 positive result to determine if admission was due to COVID-19. Laboratory test results database, that includes the microbiology Database is also available. Information on vaccination is available at the Vaccination Registry and the Administrative record database contains the sociodemographic data. Primary healthcare records allow to extract comorbidities for adjusting the estimates.

In **Norway**, information sources used for this study cover approximately 4.3 million inhabitants, around 1 million over 65 years of age. Hospitalization data is extracted from the Norwegian Intensive Care and Pandemic Registry, where Hospitalization with Covid-19 as main cause is available. Information on vaccination is available at SYSVAK – National Immunisation Register. Sociodemographic information, including country of birth is available at the National Population Register, information on comorbidities is extracted from the Norwegian Patient Registry (NPR): individual level data from all public specialist health-care services in Norway, and residents in nursing homes can be identified through the Institution register from Norwegian Labour and Welfare Administration.

Successful linkage between the vaccination database and the administrative population database was reported for close to 100% in all four study sites.

2. Study design

A retrospective cohort study using data collected in electronic health records databases with individual deterministic linkage. The risk of occurrence of study outcomes will be compared between individuals with different vaccination status.

3. Study population

The study population includes individuals in the National Vaccination Plan and/or the reference population registries fulfilling the following criteria during the different study periods:

- Resident in the EU/EEA country performing the study (not excluded in Portugal)
- Not resident in nursing homes
- Belonging to the group who was universally vaccinated as recommended by age.
- No previous infection: No previous positive SARS-CoV-2 test recorded at the date of follow-up period (first day of follow-up for each individual).

4. Definitions

4.1. Exposure: Vaccination status

The vaccination status is based on vaccine doses administered up to the date in which vaccination status is assessed (as a time-changing exposure), and individual will be classified as follows:

- Non-vaccinated: has not received any vaccine dose.
- Complete vaccination with primary series of COVID-19 vaccines: individuals who received the primary series of COVID-19 vaccine doses defined as one dose of Ad26.COV2.S (Janssen) vaccine or two doses of ChAdOx1-S (Oxford/Astra Zeneca), BNT162b2 (Pfizer-BioNTech) or mRNA-1273 (Moderna), or combination of any of the three vaccines. Doses should be administered a minimum of 19 days apart. This status is achieved 7 days (if Pfizer) or 14 days (for all other vaccines) after the date of administration of the dose of complete vaccination.
- Complete vaccination with first booster COVID-19 vaccine dose: individuals who received an additional dose of BNT162b2, mRNA-1273 or ChAdOx1-S at least 3 months after date of

complete vaccination primary series (as defined above, including the induction period of 7 days). This status is achieved 14 days after the date of administration of the booster dose.

Induction period for each dose will be considered as separate exposure category. Any individual who received at least one dose of vaccine but do not fulfil the definition of complete vaccination with primary series of COVID-19 vaccines will be considered incompletely vaccinated. This category will be analysed, but will not be reported as an Exposure category for this study.

Individuals who received the second dose within less than 19 of the first vaccine dose, or that receives a subsequent vaccine dose (any vaccine dose after complete vaccination) that does not fulfil the definition criteria of a booster will be considered information errors and be dropped from the risk set. Also, persons with vaccine brands or vaccination schedules not included in the national vaccination programme or unknown will be dropped.

Time since booster vaccination

Time since booster dose administration (plus induction period) was computed at each point in time by constructing a time-dependent variable between date of last booster dose administration + induction period and the assessment date. Time since booster was categorized in three periods, although the number of individuals in the last category will be scarce in the first months of this study:

- From 7 or 14 days up to ≤84 days (i.e. ≤ 11 weeks)
- days 85 – 168, both included (i.e. weeks 12-23)
- ≥169 days (i.e. ≥24 weeks)

4.2. Outcome: Hospitalisation due to COVID-19

Hospitalisation due to COVID-19: laboratory-confirmed infection with admission to hospital 24 hours before (48hours in Denmark) or up to 3 weeks after the positive test or symptoms onset (2 weeks in Denmark), in which admission or discharge criteria is compatible with SARI (based on similar criteria as in SARI surveillance, ICD coding or similar).

Table. Definition used for COVID-19 hospitalization in the four study sites.

Portugal	Denmark	Navarre	Norway
Admission for at least 24h with COVID-19 as the primary diagnosis (ICD10 code U07.1) at discharge	Admission for at least 12 hours with specific COVID-19 diagnosis at discharge	Confirmed admission due to COVID-19 according to a Public Health medical doctor revision of all hospitalised patients with a COVID-19 positive result.	Hospitalised with Covid-19 as main cause

4.3. Other variables

Age group

Age will be calculated at the beginning of each study period using the date of birth, and categorised into 5-years bins to adjust models. For reporting stratified results by age-group the following groups will be used 65-79, 80+. Alternative age groups may be discussed upon needs.

4.5. Estimation of vaccine effectiveness

Groups to be compared

The analysis will be done in people classified as not having previous SARS-CoV-2 infection. Vaccine effectiveness will be estimated by comparing different exposure groups to fulfil all the study objectives. The relevant comparisons include:

1. Exposed group = complete vaccination with primary series of COVID-19 vaccines; Reference group = unvaccinated. Stratified by age group: 65-79, 80-110
2. Exposed group = complete vaccination with primary series of COVID-19 vaccines + first booster; Reference group = unvaccinated. Stratified by age group: 65-79, 80-110.
3. Exposed group = complete vaccination with primary series of COVID-19 vaccines + first booster < 84 days ago; Reference group = unvaccinated. Stratified by age group: 65-79, 80-110.
4. Exposed group = complete vaccination with primary series of COVID-19 vaccines + first booster 85 – 168 days ago; Reference group = unvaccinated. Stratified by age group: 65-79, 80-110
5. Exposed group = complete vaccination with primary series of COVID-19 vaccines + first booster ≥169 days ago; Reference group = unvaccinated. Stratified by age group: 65-79, 80-110

It is recommended that contrasts that use the same reference group are performed using a single model with exposures defined by categories of a single variable. For instance, group comparisons numbered as 1 and 2 above could be done in a single model, with a variable that equals 0 for non-vaccinated, 1 for complete vaccination with primary series and 2 for first booster. Similarly, contrasts 3, 4 and 5 should be performed in a single model.

Subgroup analyses

Estimations, both crude and adjusted will be performed separately in two age groups: 65-79 and 80+ years of age, and disaggregated by time since the booster dose, as previously defined.

Crude vaccine effectiveness

Each individual will enter the study in the different exposure groups on the date they are first classified into that group. This will be date of the beginning of the study, except for individuals that change exposure groups throughout follow-up, which will be censored without event in the group that they leave and are recorded as a delayed entry in the group where they are newly classified. End of follow-up will be established at the time of occurrence of any reason for censoring, and will be marked as event=1 if the reason for censoring is the event of interest, or event=0 otherwise. The time of start and end and whether the follow-up ended in event or not will be provided to the survival command.

Vaccine effectiveness will be estimated using hazard ratio (HR) of defined outcome(s) in individuals with different exposure categories, as defined above, within the population study. Survival Cox models for the estimation of HRs will be fit with calendar time as the underlying time scale, thus assigning time 0 to the first day of the observation period.

Crude VE = (1-HR) x 100

Adjusted vaccine effectiveness

The regression to estimate HR will be adjusted by fixed or time-changing confounders, as appropriate, and as previously defined. First, partially adjusted HR will be estimated, adjusting by age group (5 year-bins), sex and region in the country, if appropriate. Second, a fully adjusted HR or RR estimate will be produced adjusting by variables related to socioeconomic condition, comorbidities and health-seeking behaviour, as relevant at each study-site.

Table. Adjustment variables in the four study sites

	Portugal	Denmark	Navarre	Norway
Age groups	Adjustment for age as a categorical variable in the following groups: 65-69, 70-74, 75-79, and 80-84, 85-89, 90-94, 95+	Adjustment for age as a categorical variable in the following groups: 65-69, 70-74, 75-79 and 80-84, 85-89, 90+	Adjustment for age as a categorical variable in the following groups: 65-69, 70-74, 75-79 and 80-84, 85-89, 90+	Natural cubic splines using knots on P25, P50 and P75 of the distribution of ages within each analysis.
Comorbidities	Yes (number of comorbidities)	Yes/no category based on ICD-10 codes. Primary and secondary diagnoses retrieved from the Danish National Patient Registry.	Immunosuppression, diabetes, cancer, cardiovascular disease, COPD, asthma, renal, cirrhosis, stroke, dementia, severe obesity	Categorised into a factor with 3 levels (no comorbidities, medium risk of severe covid and high risk of severe covid). These are combinations of ICD-10 diagnoses.
Country of residence / country of birth / nationality	no	Only Danish residents are included. No restrictions on nationality or country of birth. No adjustment for this.	Country of birth (Spain/other)	Country of birth, three-level factor: Norway, outside Norway and unknown.
Deprivation index or similar	yes	no	No	Measure of crowded living conditions (2 level factor) – a combination of number of rooms and area pr resident.
Time / calendar	yes	Calendar time was used as the underlying time scale	Calendar time was used as the underlying time	Cox models with explicit time.

		in the Cox regression, however no stratification by week.	scale in the Cox regression	
Region / Health region / Geographic level	yes	HR adjusted for residency in the 5 geographical regions of Denmark (EU NUTS-2 regions)	No	Counties (11 levels).
Other vaccines uptake in the last four years (e.g., influenza, PCV7, PCV10, PCV13, PPV23)	yes	Not possible	No	No.
Number of COVID-19 tests in 2020-2022	yes	Not possible using this as a proxy for health seeking behaviour does not make sense in DK, since people tested to be able to socialize.	No	No.
Other variables	Sex	Sex	Sex, functional dependence	Sex

Methods for pooling estimates

The crude effect, the basic adjusted effect (age, sex, region) and the fully adjusted effect (adding the rest of available covariates), will be compared to assess the degree of confounding by different factors. The fully adjusted estimate was pooled across sites to draw a single estimate.

Country-specific HRs and standard errors for the effect of COVID-19 vaccination obtained from the study studies, were combined using meta-analysis methods, both using fixed-effects and for random-effects.

In the fixed-effects approach, a weighted average of the sites' estimate was computed, together with its 95% Confidence Interval. This has the advantage of not being influenced by the low number of study sites included in the pilot phase.

The approach incorporating random effects was more plausible, since both measured and unmeasured country-specific factors are expected to influence vaccine effectiveness. However, the low number of sites included in this pilot makes this approach less efficient. Moreover, it provides the average effect between studies with relatively low influence of the size of the different studies (as compared to how they influence pooled results in the fixed-effects model), thus representing to a lower degree the average effect taking into account the size of the country reporting. Under the random-effects, the country-specific exposure-disease effects (HRs) were weighted by the inverse of their marginal variances (generic inverse variance method). The marginal variance is the sum of the individual study-specific variances and the variance of the random study effects (τ2). This gives the pooled HR and a standard error. We calculated the

confidence interval around the pooled effect (the range of values that contain the true average HR with 95% certainty). $\tau 2$ and I2 were used to describe between-studies heterogeneity, along with the p-value of the heterogeneity test. Potential factors or specific pilot sites characteristics that could be the source of qualitative heterogeneity were described and discussed.

The country-specific HR and their confidence intervals, along with the pooled HR, were presented graphically in a forest plot. Pooled estimates were obtained overall for each of the exposure categories and subgroups outlined in this protocol. For each pooled estimate, only sites contributing to that estimate were used.

Supplement S2. Number of events and person-months of follow-up in the fixed-effects model, in each of the analyses, by site.

Age	Period	Vaccination status	Variable	Total	Denmark	Navarra	Norway	Portugal
65-79	Oct'21 - Nov'21	Non-vaccinated	Events	229	84	11	65	69
			Person-months	311,465	51,866	8,028	64,003	187,568
		Complete vaccination	Events	769	330	50	164	225
			Person-months	5,430,697	1,428,050	144,064	1,363,302	2,495,281
		Complete vaccination + booster	Events	48	34	0	13	1
			Person-months	132,388	65,96	5,556	38,589	22,283
	Nov'21 - Dec'21	Non-vaccinated	Events	463	143	29	131	160
			Person-months	295,571	48,948	7,402	58,382	180,839
		Complete vaccination	Events	1,141	394	79	198	470
			Person-months	3,627,044	873,947	98,342	1,027,320	1,627,435
		Complete vaccination + booster	Events	169	97	16	30	26
			Person-months	1,199,370	353,348	43	302,518	500,505
	Dec'21 - Jan'22	Non-vaccinated	Events	535	152	56	118	209
			Person-months	280,857	45,275	6,929	54,957	173,696
		Complete vaccination	Events	766	210	64	100	392
			Person-months	1,261,165	216,077	35,33	430,957	578,801
		Complete vaccination + booster	Events	597	249	79	72	197
			Person-months	3,623,380	1,029,656	102,407	894,115	1,597,202
	Jan'22 - Feb'22	Non-vaccinated	Events	463	117	46	88	212
			Person-months	269,006	41,81	6,467	51,878	168,851
		Complete vaccination	Events	515	118	19	74	304
			Person-months	386,221	37,329	7,396	91,353	250,143
		Complete vaccination + booster	Events	1,429	748	80	282	319
			Person-months	4,981,833	1,395,084	124,425	1,226,582	2,235,742
	Feb'22 - Mar'22	Non-vaccinated	Events	362	118	0	125	119
			Person-months	251,505	38,872	0	50,405	162,228
		Complete vaccination	Events	334	87	0	127	120
			Person-months	261,264	28,681	0	66,438	166,145
		Complete vaccination + booster	Events	1,972	975	0	693	304
			Person-months	4,783,200	1,280,532	0	1,241,080	2,261,587

Age	Period	Vaccination status	Variable	Total	Denmark	Navarra	Norway	Portugal
80+	Oct'21 - Nov'21	Non-vaccinated	Events	130	46	2	35	47
			Person-months	147,143	16,035	2,503	24,818	103,786
		Complete vaccination	Events	652	238	21	190	203
			Person-months	1,561,711	321,053	52,223	413,051	775,384
		Complete vaccination + booster	Events	38	13	7	7	11
			Person-months	308,135	75,86	9,43	50,494	172,351
	Nov'21 - Dec'21	Non-vaccinated	Events	275	78	12	67	118
			Person-months	140,295	15,09	2,275	22,277	100,652
		Complete vaccination	Events	751	268	37	179	267
			Person-months	716,577	144,097	23,415	236,732	312,333
		Complete vaccination + booster	Events	181	71	22	16	72
			Person-months	1,124,695	237,346	35,058	201,936	650,356
	Dec'21 - Jan'22	Non-vaccinated	Events	453	89	49	77	238
			Person-months	135,547	13,788	2,127	20,514	99,118
		Complete vaccination	Events	620	148	50	74	348
			Person-months	262,138	34,242	4,485	75,28	148,13
		Complete vaccination + booster	Events	884	243	123	49	469
			Person-months	1,752,544	386,55	52,734	361,553	951,707
	Jan'22 - Feb'22	Non-vaccinated	Events	546	123	51	80	292
			Person-months	129,959	12,698	1,993	19,054	96,215
		Complete vaccination	Events	544	91	32	65	356
			Person-months	148,922	11,611	1,946	30,4	104,964
		Complete vaccination + booster	Events	1,938	704	134	257	843
			Person-months	1,901,312	433,12	52,973	405,069	1,010,151
	Feb'22 - Mar'22	Non-vaccinated	Events	433	127	0	138	168
			Person-months	123,091	11,918	0	18,093	93,081
		Complete vaccination	Events	399	89	0	121	189
			Person-months	117,721	9,254	0	26,094	82,372
		Complete vaccination + booster	Events	2,493	1,026	0	701	766
			Person-months	1,819,768	415,297	0	404,468	1,000,004

Supplement S3. Detailed site estimates and pooled results using either fixed or random effects meta-analysis.

1. Detailed site estimates and pooled results with fixed and random effects meta-analysis during the observation period October 1 – November 25 2021

Estimates for overall complete and booster vaccination in the group 65-79 years

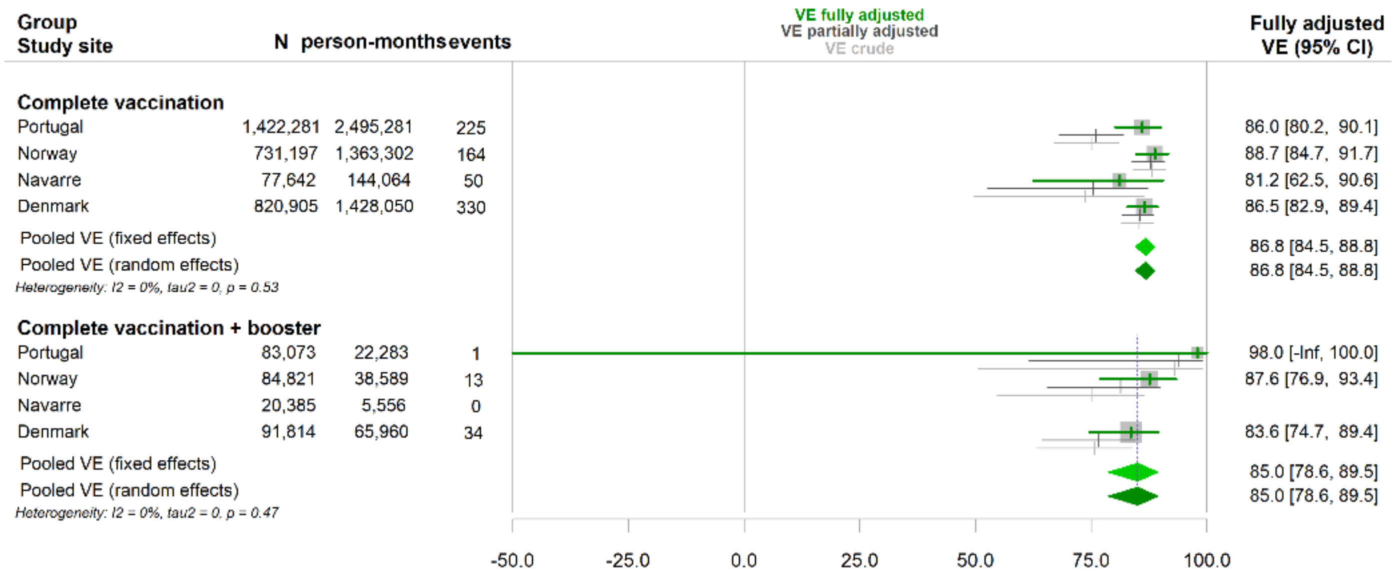

Estimates for overall complete and booster vaccination in the group 80 years

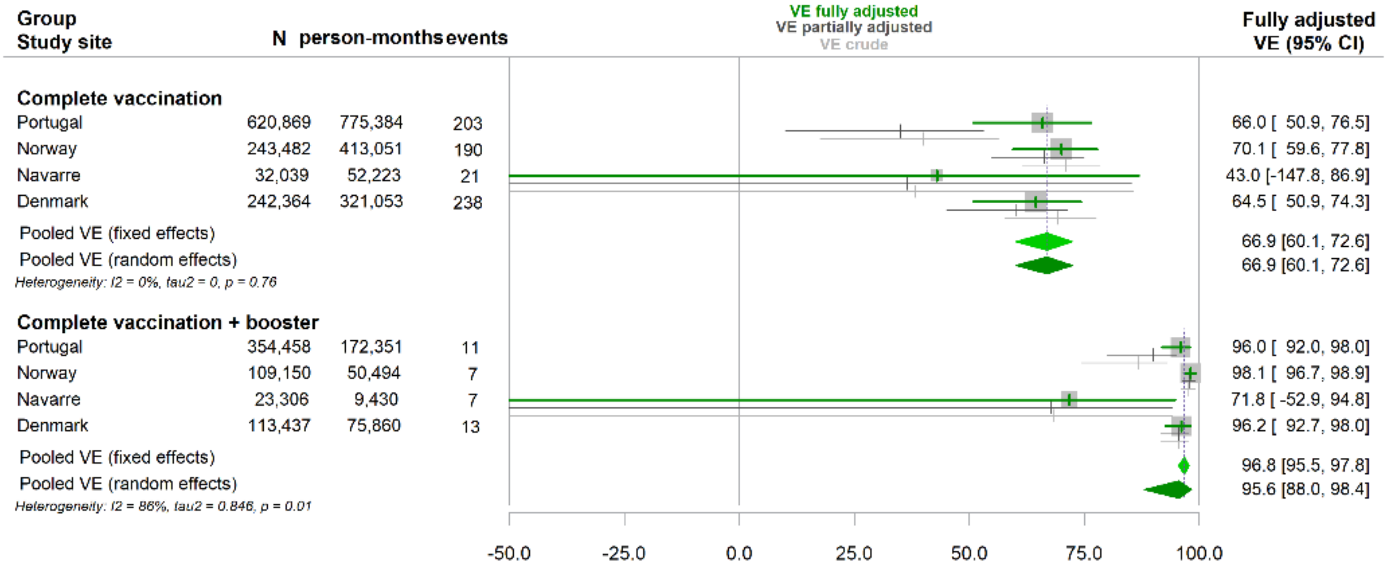

Estimates by time since booster vaccination in the group 65-79 years

Group<br>Study site	N	person-months	events	Fully adjusted VE (95% CI)
**Complete vaccination + booster <12 weeks**				
Portugal	83,073	22,283	1	98.0 [-Inf, 100.0]
Norway	96,852	38,233	12	87.4 [75.9, 93.4]
Navarre	20,385	5,556	0	
Denmark	91,811	65,948	34	83.6 [74.7, 89.4]
Pooled VE (fixed effects)				84.9 [78.3, 89.5]
Pooled VE (random effects)				84.9 [78.3, 89.5]
Heterogeneity: I2 = 0%, tau2 = 0, p = 0.51				
**Complete vaccination + booster ≥12 & <24 weeks**				
Portugal	0	0	0	
Norway	454	339	1	31.4 [-413.4, 90.8]
Navarre	0	0	0	
Denmark	20	12	0	
Pooled VE (fixed effects)				
Pooled VE (random effects)				
Heterogeneity: I2 = 0%, tau2 = 0, p = 1				
**Complete vaccination + booster ≥24 weeks**				
Portugal	0	0	0	
Norway	30	17	0	
Navarre	0	0	0	
Denmark	1	0	0	
Pooled VE (fixed effects)				
Pooled VE (random effects)				

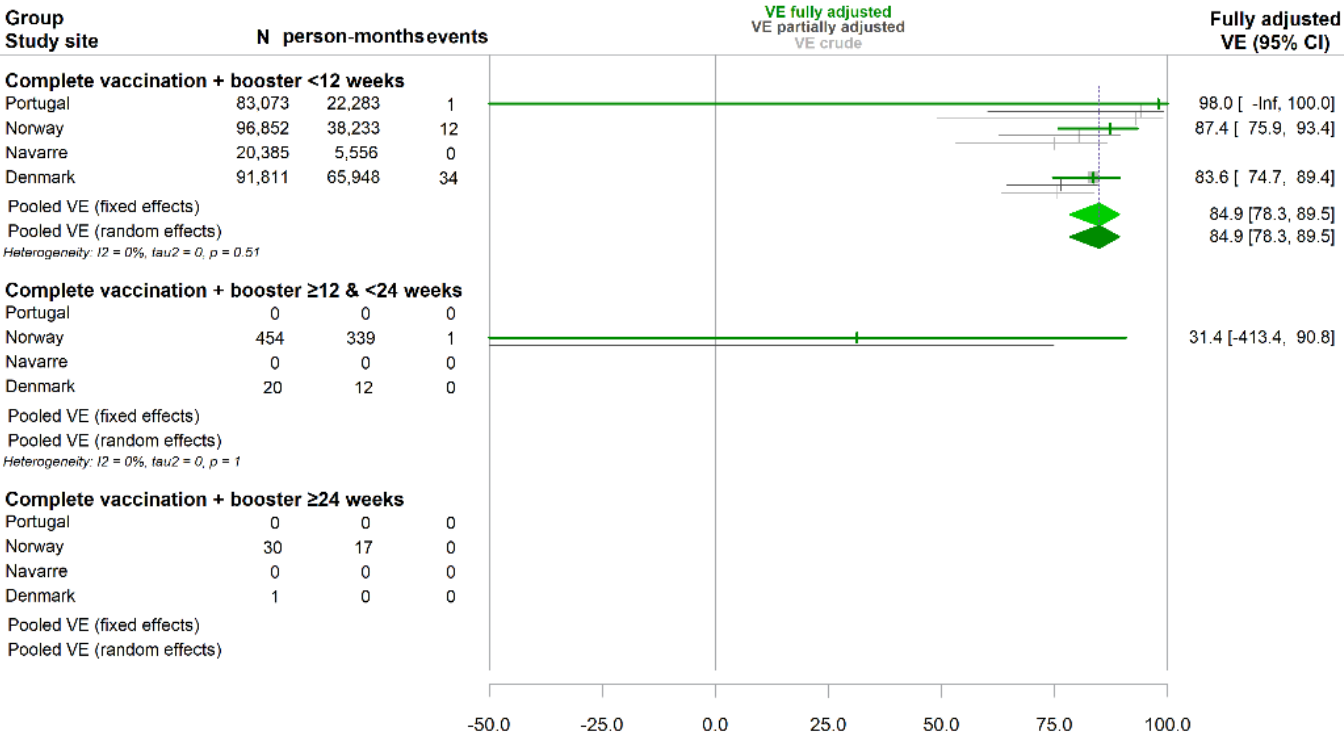

Estimates by time since booster vaccination in the group 80 years

Group<br>Study site	N	person-months	events	Fully adjusted VE (95% CI)
**Complete vaccination + booster <12 weeks**				
Portugal	354,458	172,351	11	96.0 [91.5, 98.1]
Norway	110,211	50,389	6	94.2 [85.9, 97.6]
Navarre	23,305	9,428	7	71.5 [-54.0, 94.7]
Denmark	113,435	75,855	13	96.2 [92.7, 98.0]
Pooled VE (fixed effects)				95.2 [92.7, 96.8]
Pooled VE (random effects)				94.4 [88.2, 97.3]
Heterogeneity: I2 = 63%, tau2 = 0.345, p = 0.16				
**Complete vaccination + booster ≥12 & <24 weeks**				
Portugal	0	0	0	
Norway	82	82	1	
Navarre	1	1	0	
Denmark	5	6	0	
Pooled VE (fixed effects)				
Pooled VE (random effects)				
Heterogeneity: I2 = 0%, tau2 = 0, p = 1				
**Complete vaccination + booster ≥24 weeks**				
Portugal	0	0	0	
Norway	32	23	0	
Navarre	1	1	0	
Denmark	NA	NA	NA	
Pooled VE (fixed effects)				
Pooled VE (random effects)				

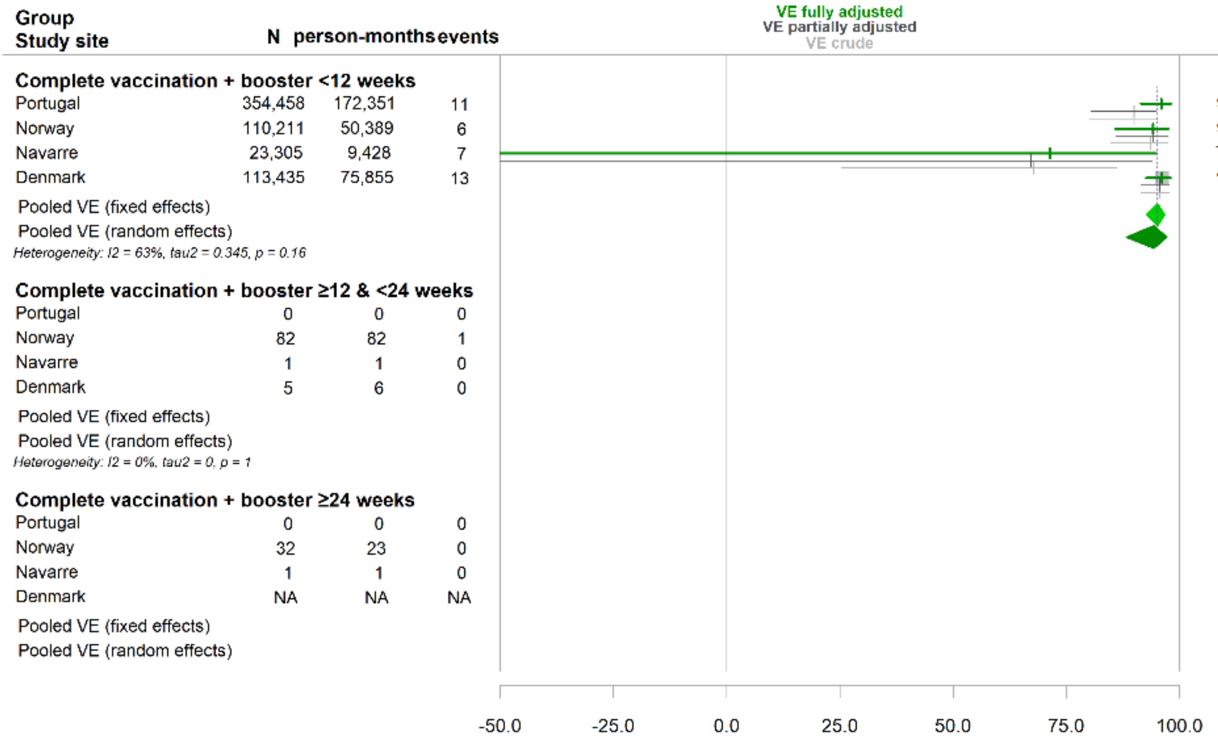

2. Detailed site estimates and pooled results with fixed and random effects meta-analysis during the observation period November 1– December 26 2021

Estimates for overall complete and booster vaccination in the group 65-79 years

Group / Study site	N	person-months	events	Fully adjusted VE (95% CI)
**Complete vaccination**				
Portugal	1,403,142	1,627,435	470	84.0 [80.2, 87.1]
Norway	722,461	1,027,320	198	91.3 [89.0, 93.1]
Navarre	77,439	98,342	79	84.1 [75.1, 89.9]
Denmark	773,274	873,947	394	79.2 [74.4, 83.1]
Pooled VE (fixed effects)				85.2 [83.3, 86.9]
Pooled VE (random effects)				85.4 [78.8, 89.9]
Heterogeneity: I2 = 88%, tau2 = 0.122, p = <0.01				
**Complete vaccination + booster**				
Portugal	979,327	500,505	26	95.0 [91.8, 96.9]
Norway	536,013	302,518	30	97.4 [96.0, 98.3]
Navarre	59,000	43,000	16	95.7 [91.7, 97.8]
Denmark	583,012	353,348	97	92.7 [90.3, 94.5]
Pooled VE (fixed effects)				94.9 [93.7, 95.8]
Pooled VE (random effects)				95.4 [92.9, 97.0]
Heterogeneity: I2 = 74%, tau2 = 0.142, p = <0.01				

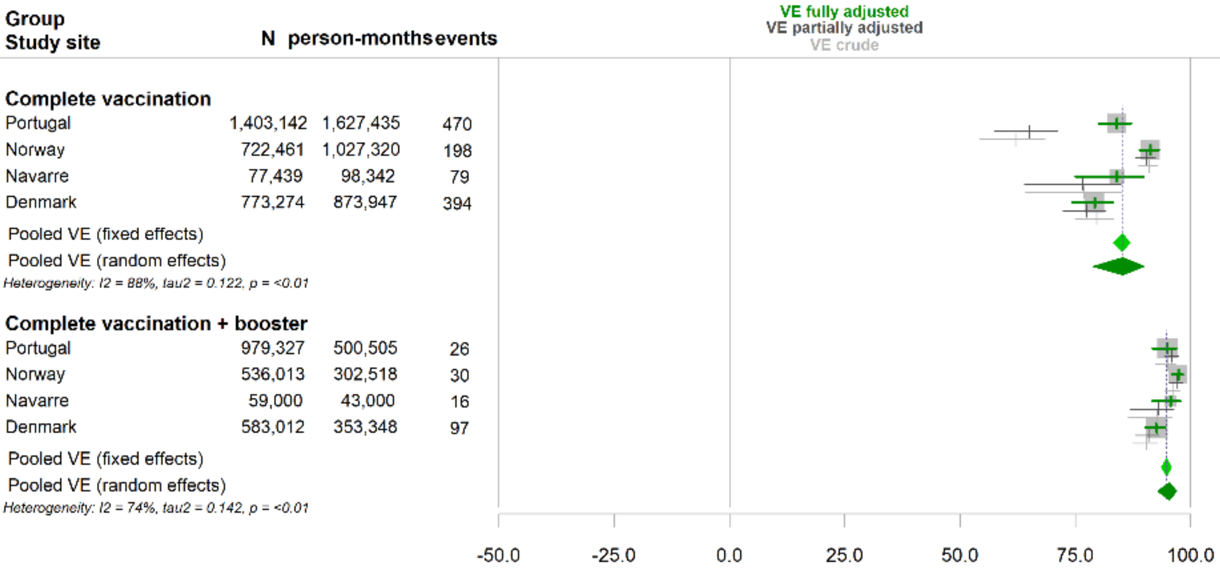

Estimates for overall complete and booster vaccination in the group 80 years

Group / Study site	N	person-months	events	Fully adjusted VE (95% CI)
**Complete vaccination**				
Portugal	405,423	312,333	267	62.0 [51.9, 70.0]
Norway	224,290	236,732	179	70.1 [59.6, 77.8]
Navarre	32,073	23,415	37	46.6 [-8.1, 73.6]
Denmark	173,081	144,097	268	50.9 [35.8, 62.5]
Pooled VE (fixed effects)				60.6 [54.3, 66.1]
Pooled VE (random effects)				60.3 [49.7, 68.7]
Heterogeneity: I2 = 54%, tau2 = 0.03, p = 0.09				
**Complete vaccination + booster**				
Portugal	524,652	650,356	72	92.0 [88.7, 94.3]
Norway	207,410	201,936	16	98.1 [96.7, 98.9]
Navarre	30,035	35,058	22	91.9 [82.9, 96.2]
Denmark	214,280	237,346	71	95.4 [93.6, 96.7]
Pooled VE (fixed effects)				94.8 [93.6, 95.8]
Pooled VE (random effects)				95.2 [90.6, 97.5]
Heterogeneity: I2 = 88%, tau2 = 0.396, p = <0.01				

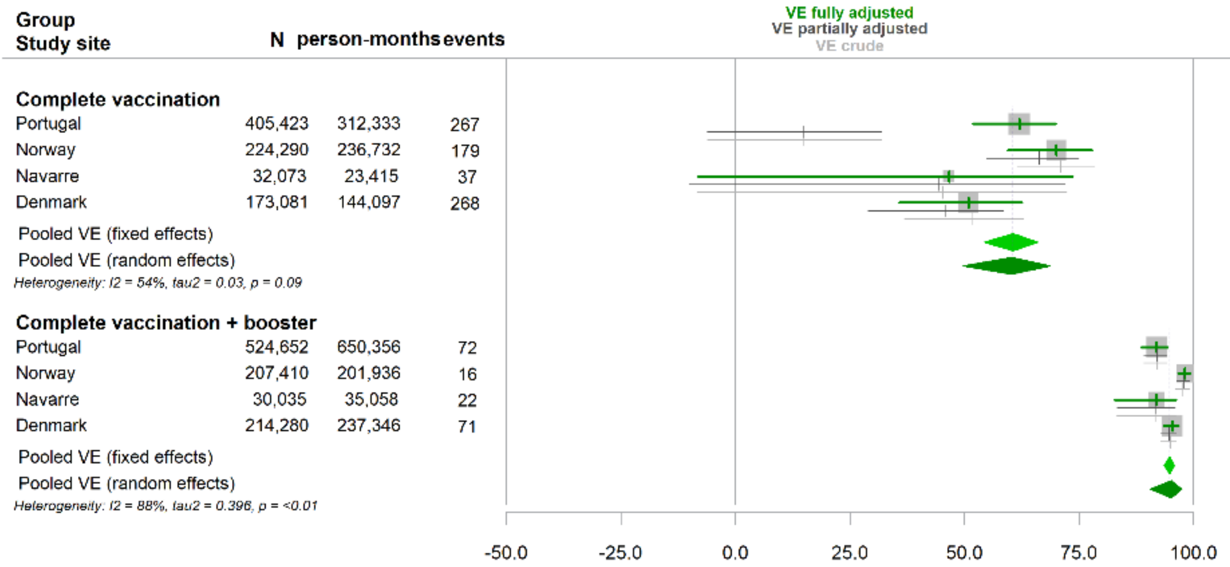

Estimates by time since booster vaccination in the group 65-79 years

Group<br>Study site	N	person-months	events	Fully adjusted VE (95% CI)
**Complete vaccination + booster <12 weeks**				
Portugal	979,327	500,493	26	95.0 [91.8, 96.9]
Norway	564,967	301,828	29	97.4 [96.0, 98.3]
Navarre	59,000	42,946	14	96.1 [92.2, 98.0]
Denmark	583,007	351,469	92	92.9 [90.5, 94.7]
Pooled VE (fixed effects)				95.0 [93.9, 95.9]
Pooled VE (random effects)				95.5 [93.1, 97.1]
Heterogeneity: I2 = 74%, tau2 = 0.142, p = <0.01				
**Complete vaccination + booster ≥12 & <24 weeks**				
Portugal	43	12	0	
Norway	1,700	637	1	79.2 [-52.8, 97.2]
Navarre	206	53	2	-9.4 [-480.6, 79.4]
Denmark	9,433	1,877	5	57.1 [-12.4, 83.6]
Pooled VE (fixed effects)				53.0 [-1.5, 78.2]
Pooled VE (random effects)				53.0 [-1.5, 78.2]
Heterogeneity: I2 = 0%, tau2 = 0, p = 0.44				
**Complete vaccination + booster ≥24 weeks**				
Portugal	0	0	0	
Norway	55	53	0	
Navarre	0	0	0	
Denmark	3	2	0	
Pooled VE (fixed effects)				
Pooled VE (random effects)				

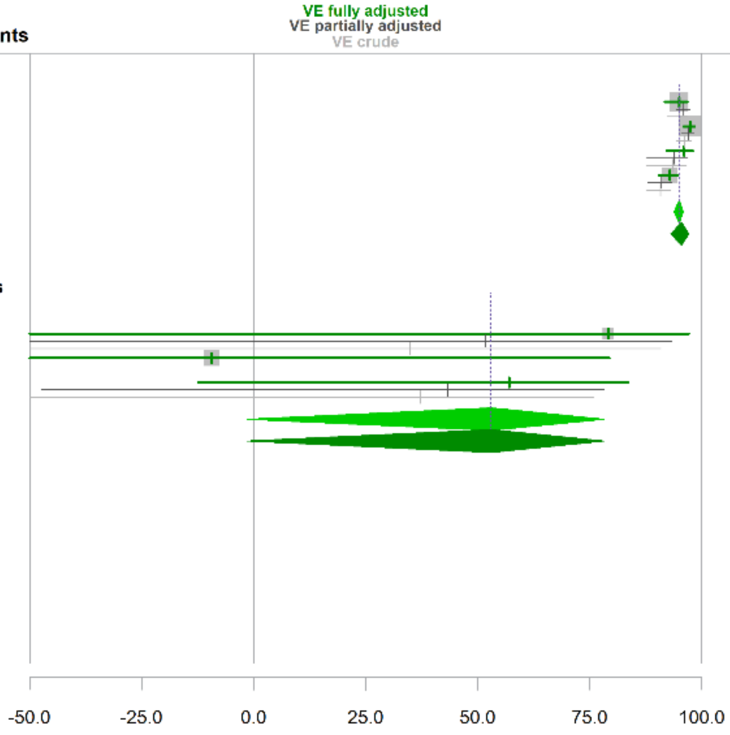

Estimates by time since booster vaccination in the group 80 years

Group<br>Study site	N	person-months	events	Fully adjusted VE (95% CI)
**Complete vaccination + booster <12 weeks**				
Portugal	524,652	650,351	72	93.0 [90.1, 95.1]
Norway	208,367	201,792	16	98.1 [96.8, 98.9]
Navarre	30,034	35,037	22	91.9 [82.8, 96.2]
Denmark	214,277	236,919	70	95.4 [93.6, 96.7]
Pooled VE (fixed effects)				95.1 [93.9, 96.0]
Pooled VE (random effects)				95.3 [91.2, 97.5]
Heterogeneity: I2 = 87%, tau2 = 0.351, p = <0.01				
**Complete vaccination + booster ≥12 & <24 weeks**				
Portugal	16	5	0	
Norway	276	94	0	
Navarre	92	19	0	
Denmark	2,355	427	1	76.9 [-77.9, 97.0]
Pooled VE (fixed effects)				
Pooled VE (random effects)				
Heterogeneity: I2 = 0%, tau2 = 0, p = 1				
**Complete vaccination + booster ≥24 weeks**				
Portugal	0	0	0	
Norway	38	50	0	
Navarre	1	2	0	
Denmark	1	0	0	
Pooled VE (fixed effects)				
Pooled VE (random effects)				

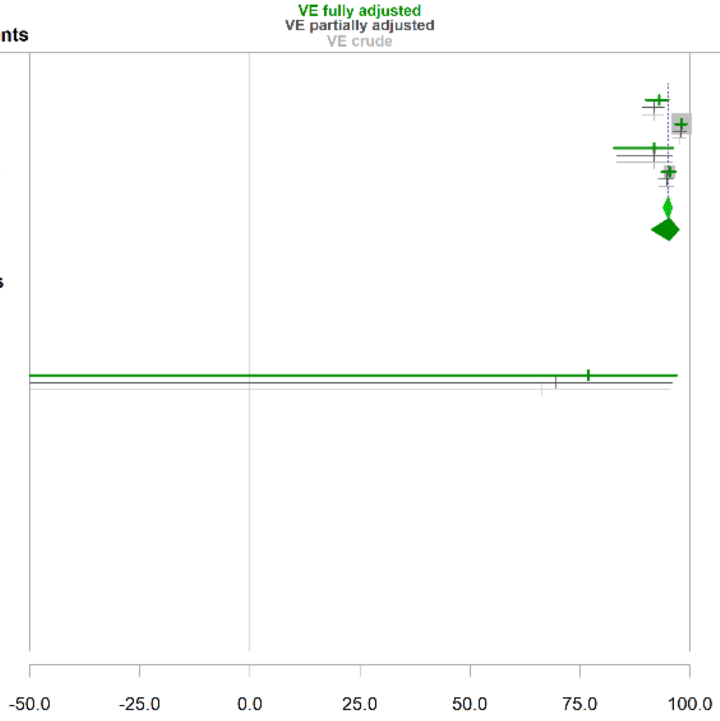

3. Detailed site estimates and pooled results with fixed and random effects meta-analysis during the observation period December 1 2021– January 252022

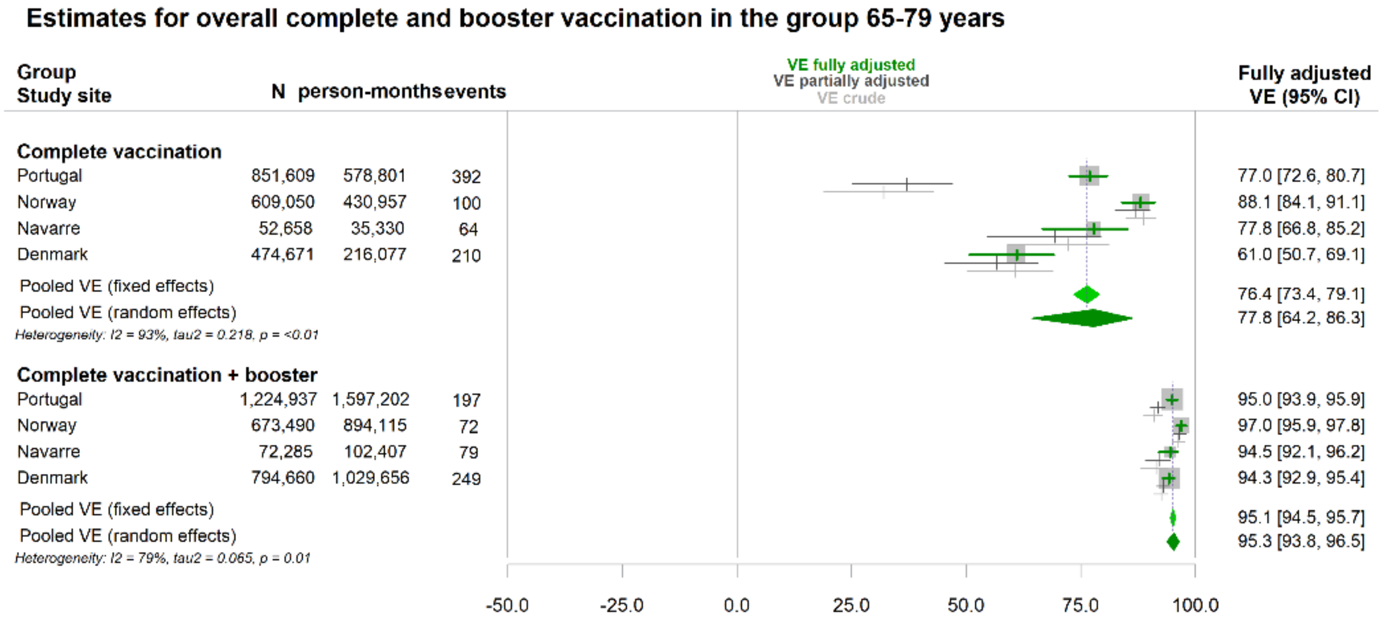

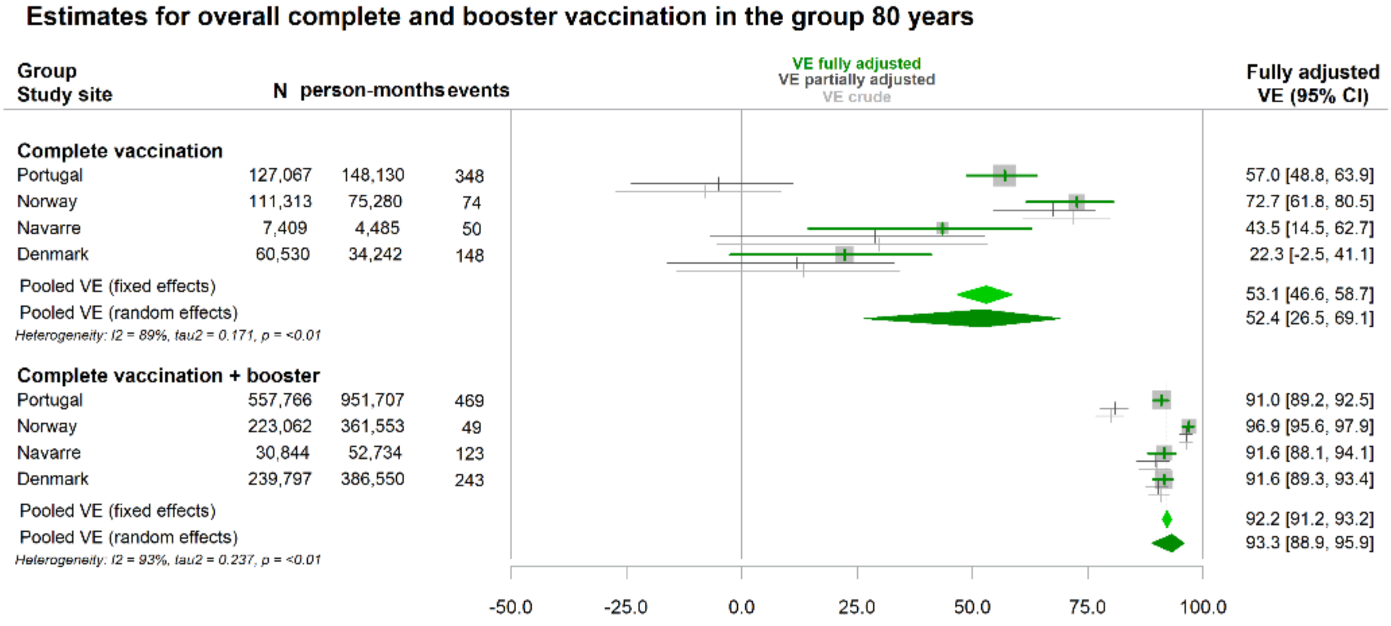

Estimates by time since booster vaccination in the group 65-79 years

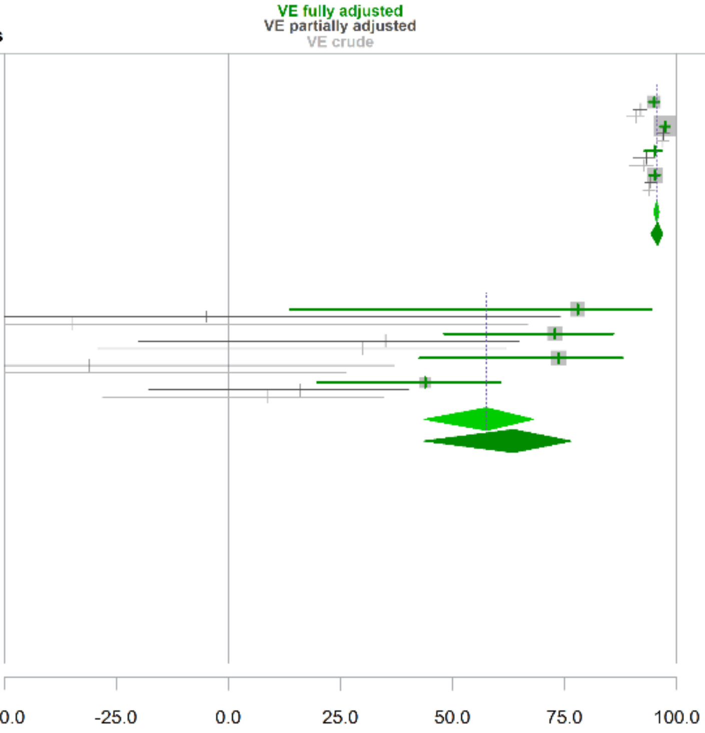

Group<br>Study site	N	person-months	events	Fully adjusted<br>VE (95% CI)
**Complete vaccination + booster <12 weeks**				
Portugal	1,224,937	1,596,404	195	95.0 [93.9, 95.9]
Norway	705,310	885,592	60	97.4 [96.4, 98.1]
Navarre	72,285	101,608	68	95.1 [92.9, 96.6]
Denmark	794,634	1,010,946	195	95.2 [94.0, 96.1]
Pooled VE (fixed effects)				95.5 [94.9, 96.1]
Pooled VE (random effects)				95.8 [94.3, 96.9]
Heterogeneity: I2 = 81%, tau2 = 0.076, p = 0.01				
**Complete vaccination + booster ≥12 & <24 weeks**				
Portugal	4,902	799	2	78.0 [13.9, 94.4]
Norway	15,617	8,338	12	72.9 [48.2, 85.8]
Navarre	3,619	798	11	73.7 [42.7, 87.9]
Denmark	27,313	18,705	54	43.9 [19.9, 60.7]
Pooled VE (fixed effects)				57.6 [43.7, 68.1]
Pooled VE (random effects)				63.6 [43.6, 76.5]
Heterogeneity: I2 = 41%, tau2 = 0.08, p = 0.09				
**Complete vaccination + booster ≥24 weeks**				
Portugal	0	0	0	
Norway	203	185	0	
Navarre	0	0	0	
Denmark	5	5	0	
Pooled VE (fixed effects)				
Pooled VE (random effects)				

Estimates by time since booster vaccination in the group 80 years

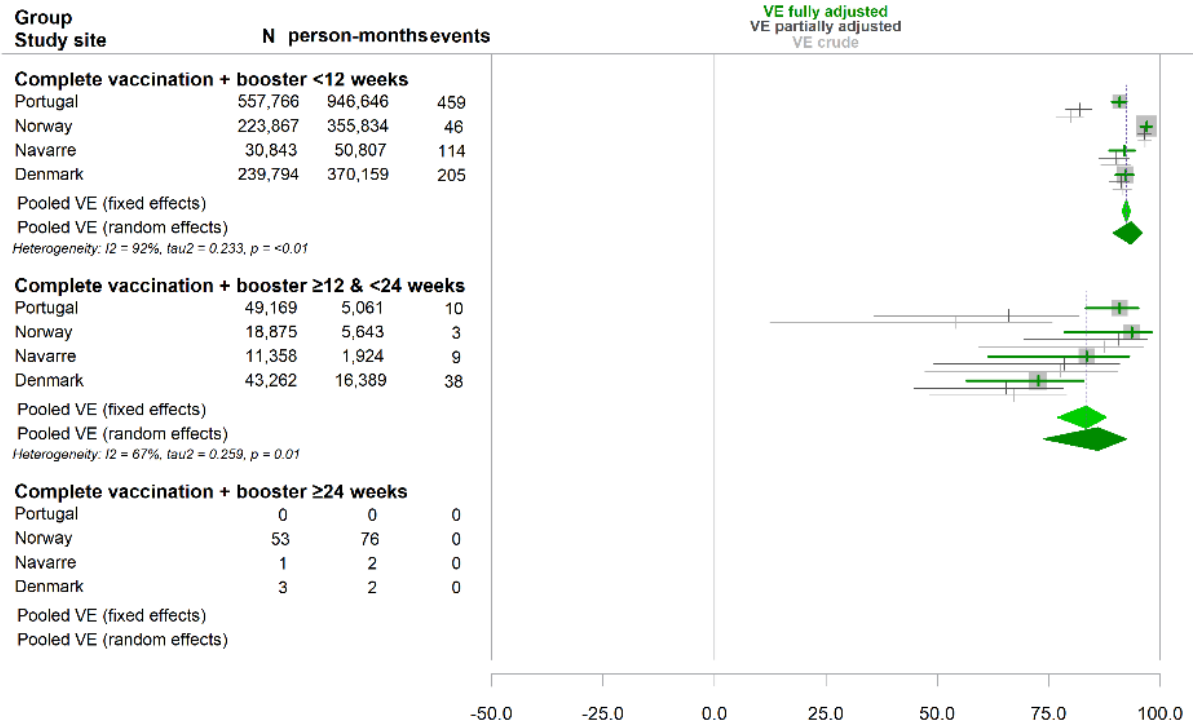

Group<br>Study site	N	person-months	events	Fully adjusted<br>VE (95% CI)
**Complete vaccination + booster <12 weeks**				
Portugal	557,766	946,646	459	91.0 [89.2, 92.5]
Norway	223,867	355,834	46	97.0 [95.7, 98.0]
Navarre	30,843	50,807	114	92.0 [88.7, 94.3]
Denmark	239,794	370,159	205	92.3 [90.1, 94.0]
Pooled VE (fixed effects)				92.5 [91.5, 93.4]
Pooled VE (random effects)				93.5 [89.4, 96.1]
Heterogeneity: I2 = 92%, tau2 = 0.233, p = <0.01				
**Complete vaccination + booster ≥12 & <24 weeks**				
Portugal	49,169	5,061	10	91.0 [83.4, 95.1]
Norway	18,875	5,643	3	93.7 [78.6, 98.1]
Navarre	11,358	1,924	9	83.6 [61.5, 93.0]
Denmark	43,262	16,389	38	72.7 [56.6, 82.8]
Pooled VE (fixed effects)				83.4 [77.0, 88.0]
Pooled VE (random effects)				86.0 [73.8, 92.5]
Heterogeneity: I2 = 67%, tau2 = 0.259, p = 0.01				
**Complete vaccination + booster ≥24 weeks**				
Portugal	0	0	0	
Norway	53	76	0	
Navarre	1	2	0	
Denmark	3	2	0	
Pooled VE (fixed effects)				
Pooled VE (random effects)				

4. Detailed site estimates and pooled results with fixed and random effects meta-analysis during the observation period January 1 – February 25 2022

Estimates for overall complete and booster vaccination in the group 65-79 years

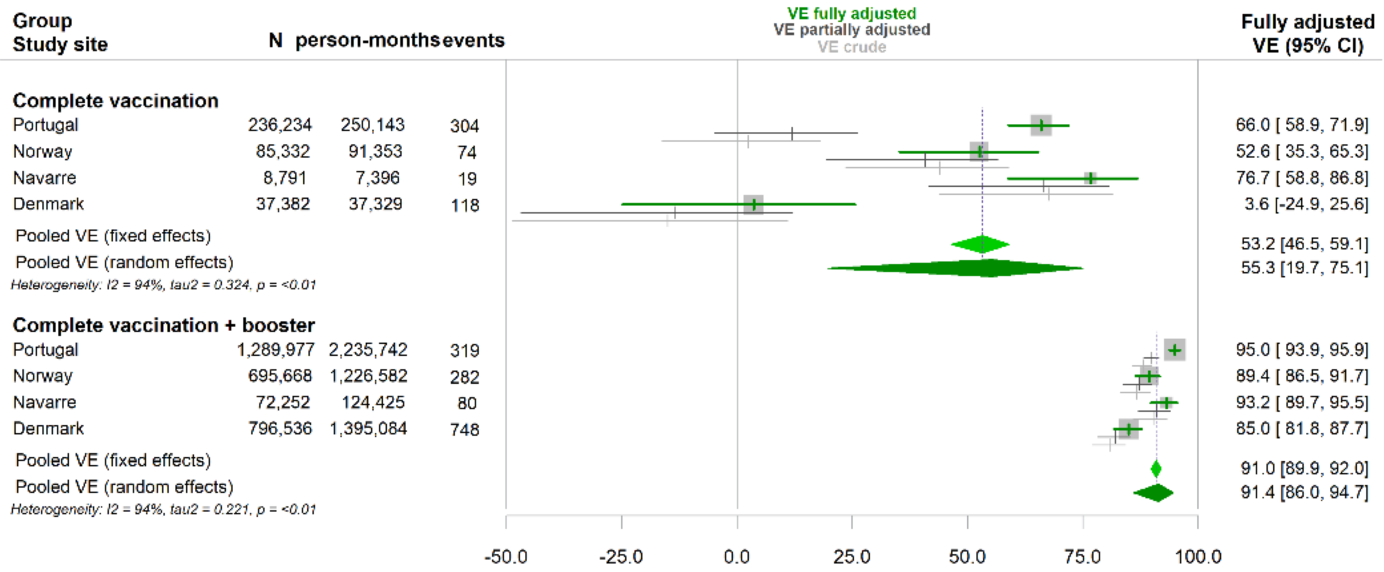

Estimates for overall complete and booster vaccination in the group 80 years

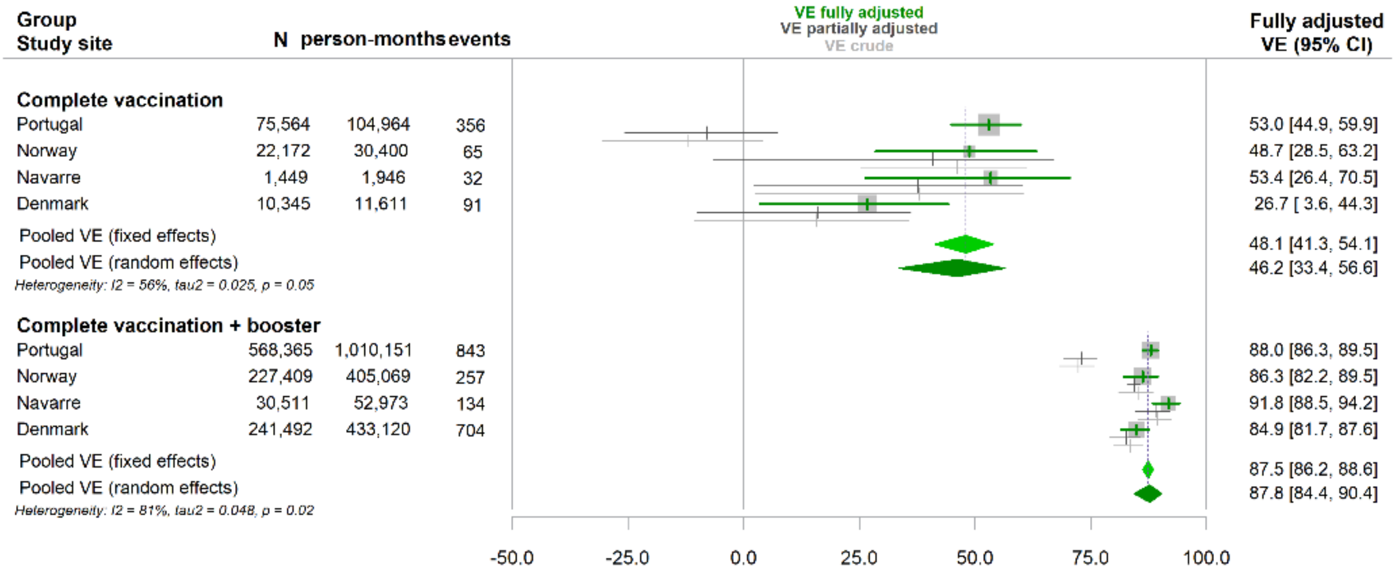

Estimates by time since booster vaccination in the group 65-79 years

Group / Study site	N	person-months	events	Fully adjusted VE (95% CI)
**Complete vaccination + booster <12 weeks**				
Portugal	1,289,883	2,180,818	291	96.0 [95.5, 96.4]
Norway	721,074	1,130,444	170	92.1 [89.7, 93.9]
Navarre	71,978	99,937	63	93.7 [90.4, 95.9]
Denmark	783,066	1,313,710	473	88.9 [86.4, 91.0]
Pooled VE (fixed effects)				94.6 [94.1, 95.1]
Pooled VE (random effects)				93.2 [89.5, 95.6]
Heterogeneity: I2 = 94%, tau2 = 0.176, p = <0.01				
**Complete vaccination + booster ≥12 & <24 weeks**				
Portugal	189,607	54,924	28	89.0 [82.9, 92.9]
Norway	256,498	96,054	112	76.1 [67.6, 82.4]
Navarre	42,973	24,488	17	89.3 [79.1, 94.5]
Denmark	129,521	81,360	275	40.4 [24.1, 53.2]
Pooled VE (fixed effects)				68.3 [62.5, 73.2]
Pooled VE (random effects)				79.1 [53.6, 90.6]
Heterogeneity: I2 = 95%, tau2 = 0.615, p = <0.01				
**Complete vaccination + booster ≥24 weeks**				
Portugal	0	0	0	
Norway	141	85	0	
Navarre	0	0	0	
Denmark	21	15	0	
Pooled VE (fixed effects)				
Pooled VE (random effects)				

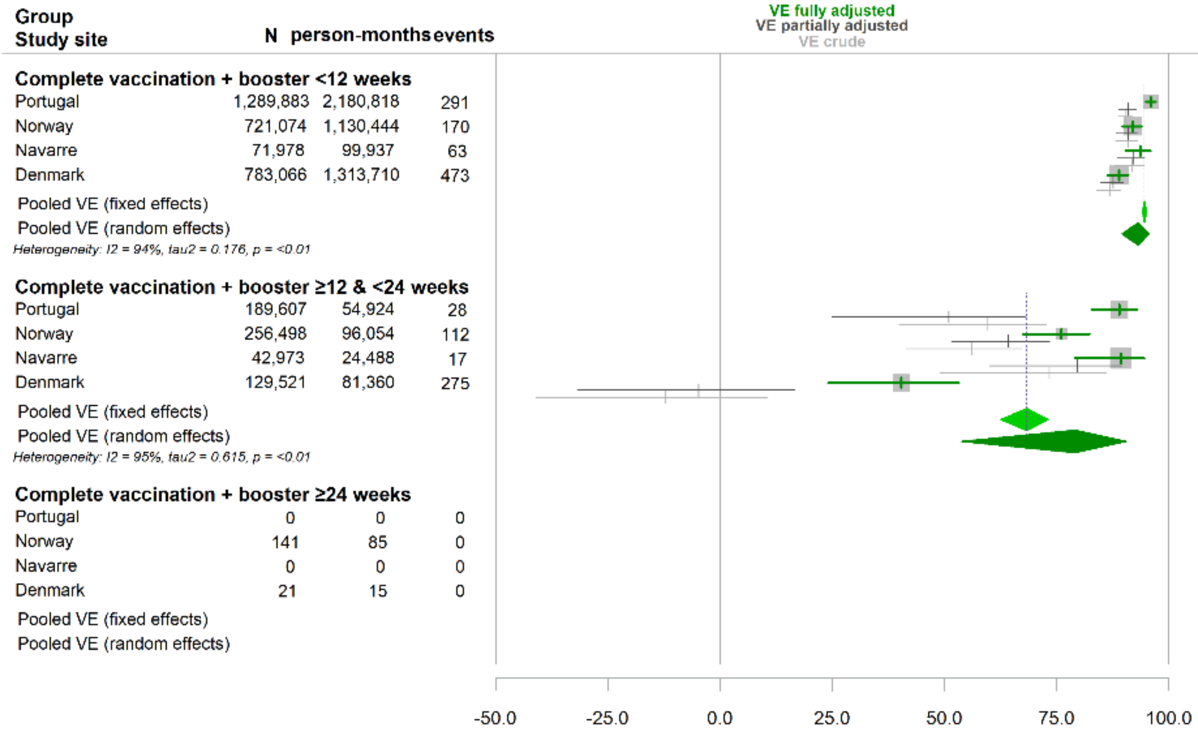

Estimates by time since booster vaccination in the group 80 years

Group / Study site	N	person-months	events	Fully adjusted VE (95% CI)
**Complete vaccination + booster <12 weeks**				
Portugal	568,336	737,269	573	89.0 [86.8, 90.8]
Norway	225,982	301,255	108	89.5 [85.7, 92.2]
Navarre	30,397	28,705	95	92.7 [89.6, 94.9]
Denmark	237,570	331,038	360	86.8 [83.7, 89.3]
Pooled VE (fixed effects)				89.0 [87.6, 90.2]
Pooled VE (random effects)				89.4 [86.7, 91.6]
Heterogeneity: I2 = 70%, tau2 = 0.037, p = 0.05				
**Complete vaccination + booster ≥12 & <24 weeks**				
Portugal	425,239	272,882	270	88.0 [85.3, 90.2]
Norway	169,781	103,778	149	82.5 [76.6, 86.9]
Navarre	27,420	24,266	39	89.2 [82.1, 93.5]
Denmark	139,434	102,078	344	78.4 [72.9, 82.8]
Pooled VE (fixed effects)				84.4 [82.3, 86.3]
Pooled VE (random effects)				84.7 [79.1, 88.8]
Heterogeneity: I2 = 80%, tau2 = 0.078, p = <0.01				
**Complete vaccination + booster ≥24 weeks**				
Portugal	0	0	0	
Norway	34	36	0	
Navarre	1	2	0	
Denmark	3	4	0	
Pooled VE (fixed effects)				
Pooled VE (random effects)				

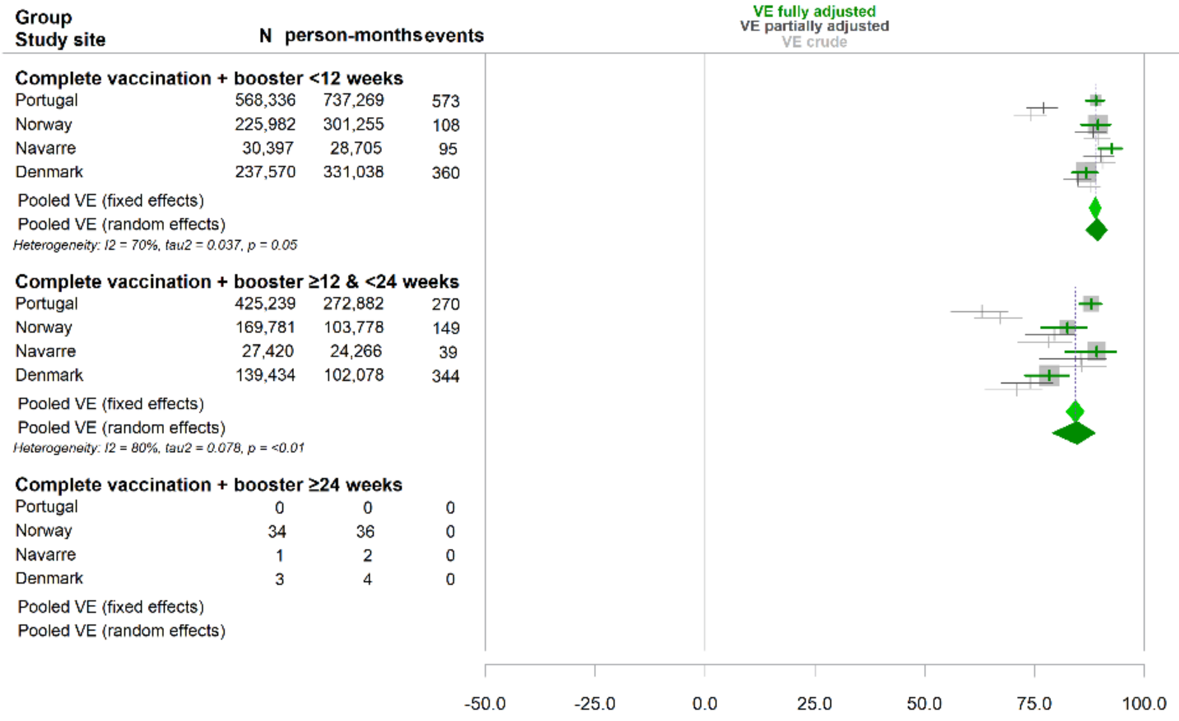

5. Detailed site estimates and pooled results with fixed and random effects meta-analysis during the observation period February 1 – March 28 2022

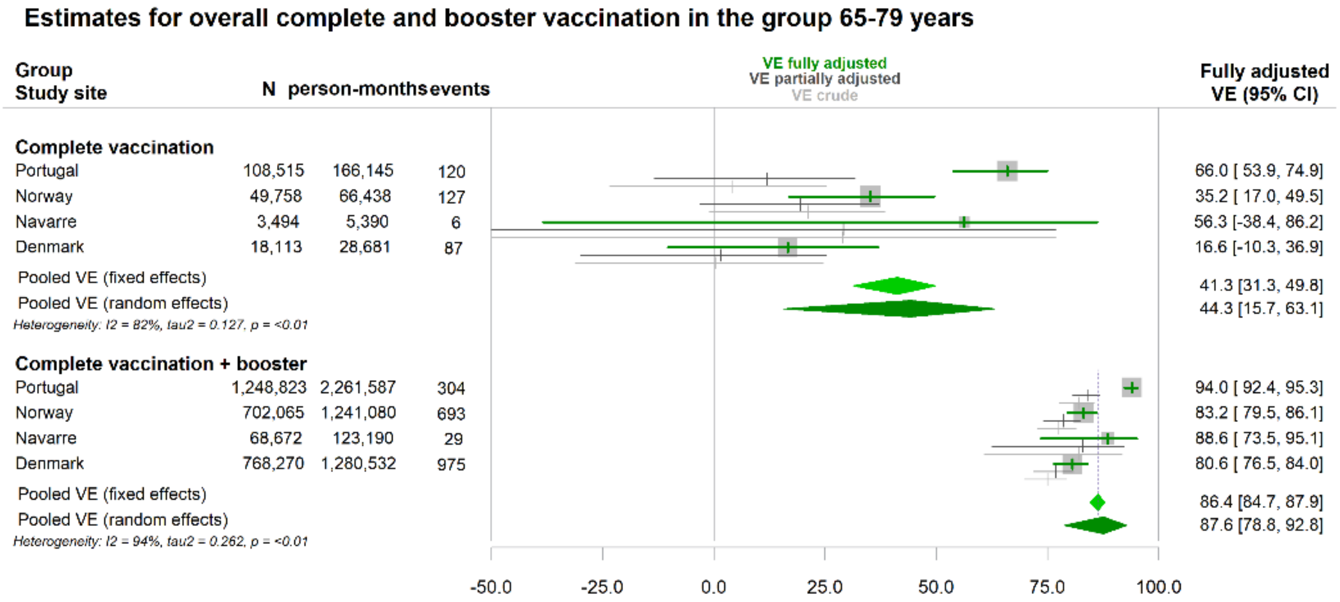

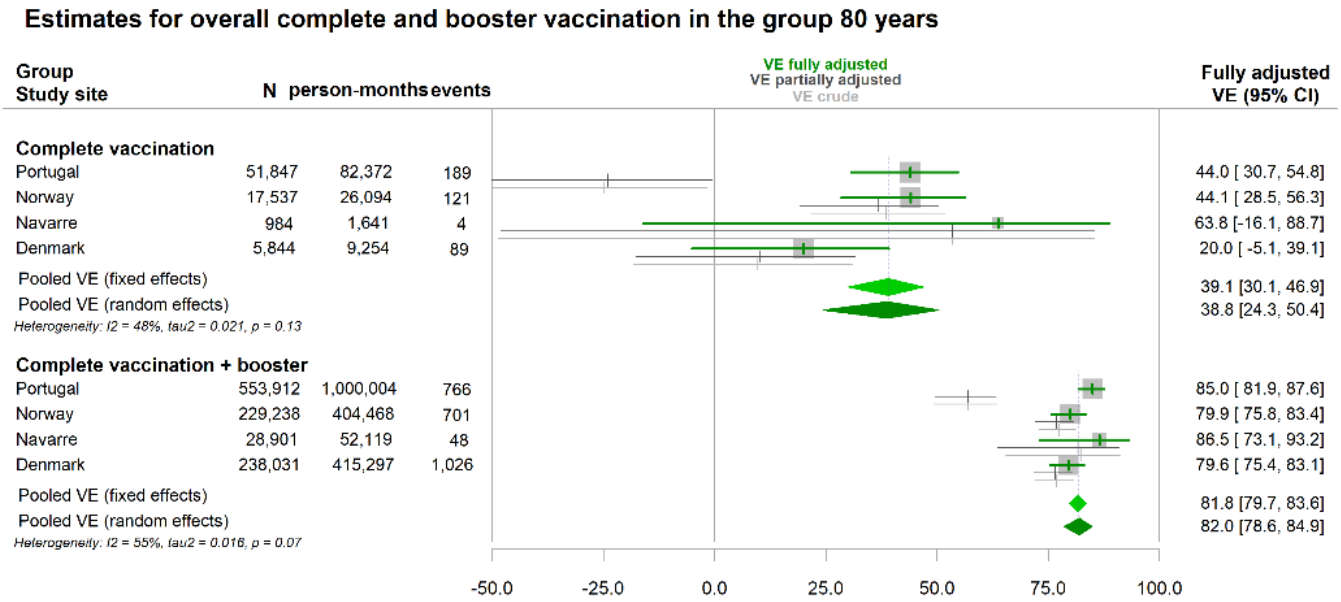

Estimates by time since booster vaccination in the group 65-79 years

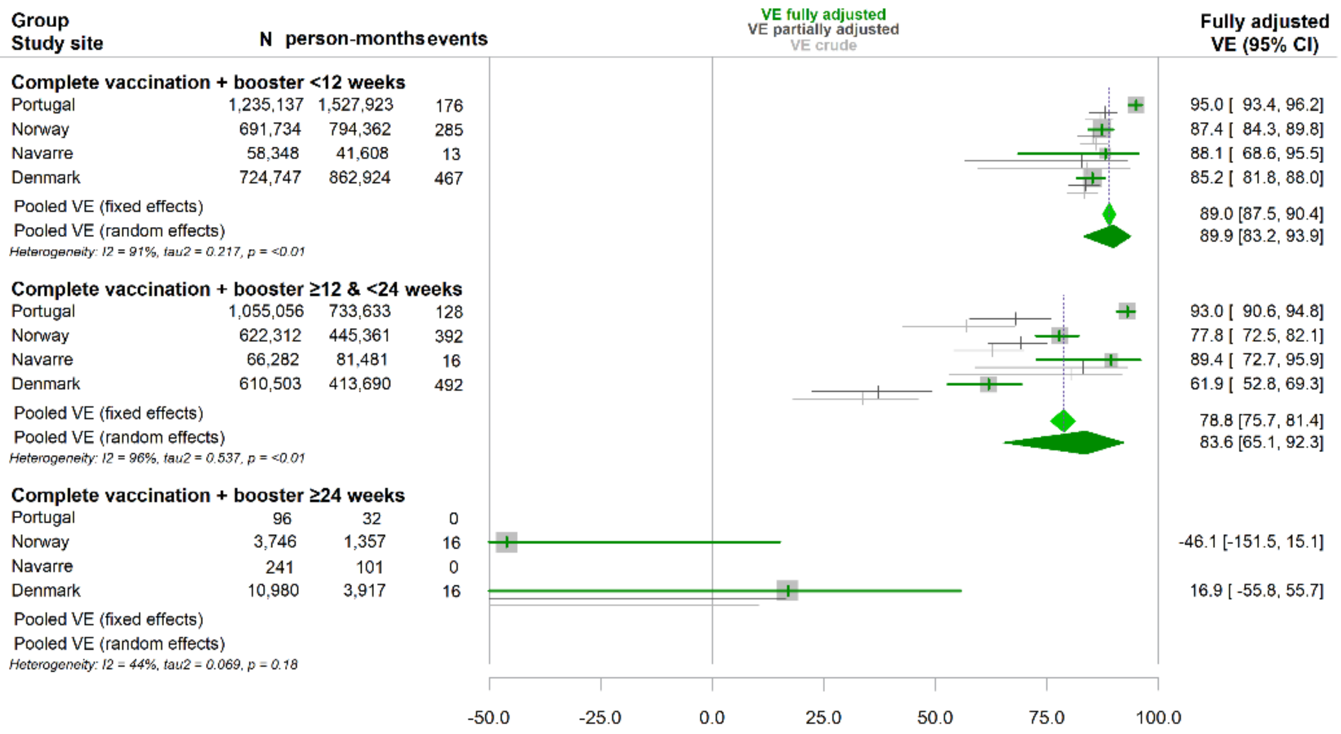

Estimates by time since booster vaccination in the group 80 years

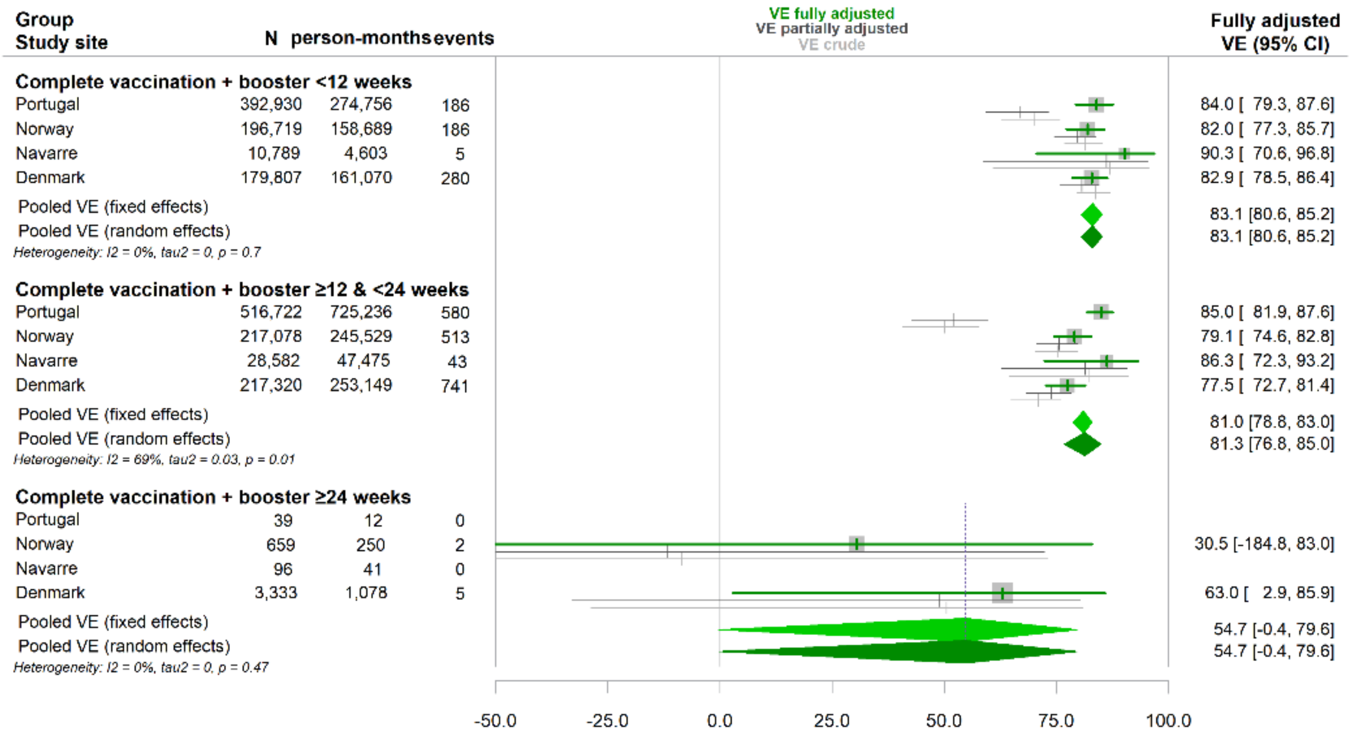

Supplement S4. Results of the sensitivity analysis using fixed-effects meta-analysis instead of random-effects. Estimated vaccine effectiveness against hospitalisation due to COVID-19 (and 95% confidence intervals), in November 2021 - March 2022, in Denmark, Portugal, Navarre and Norway.

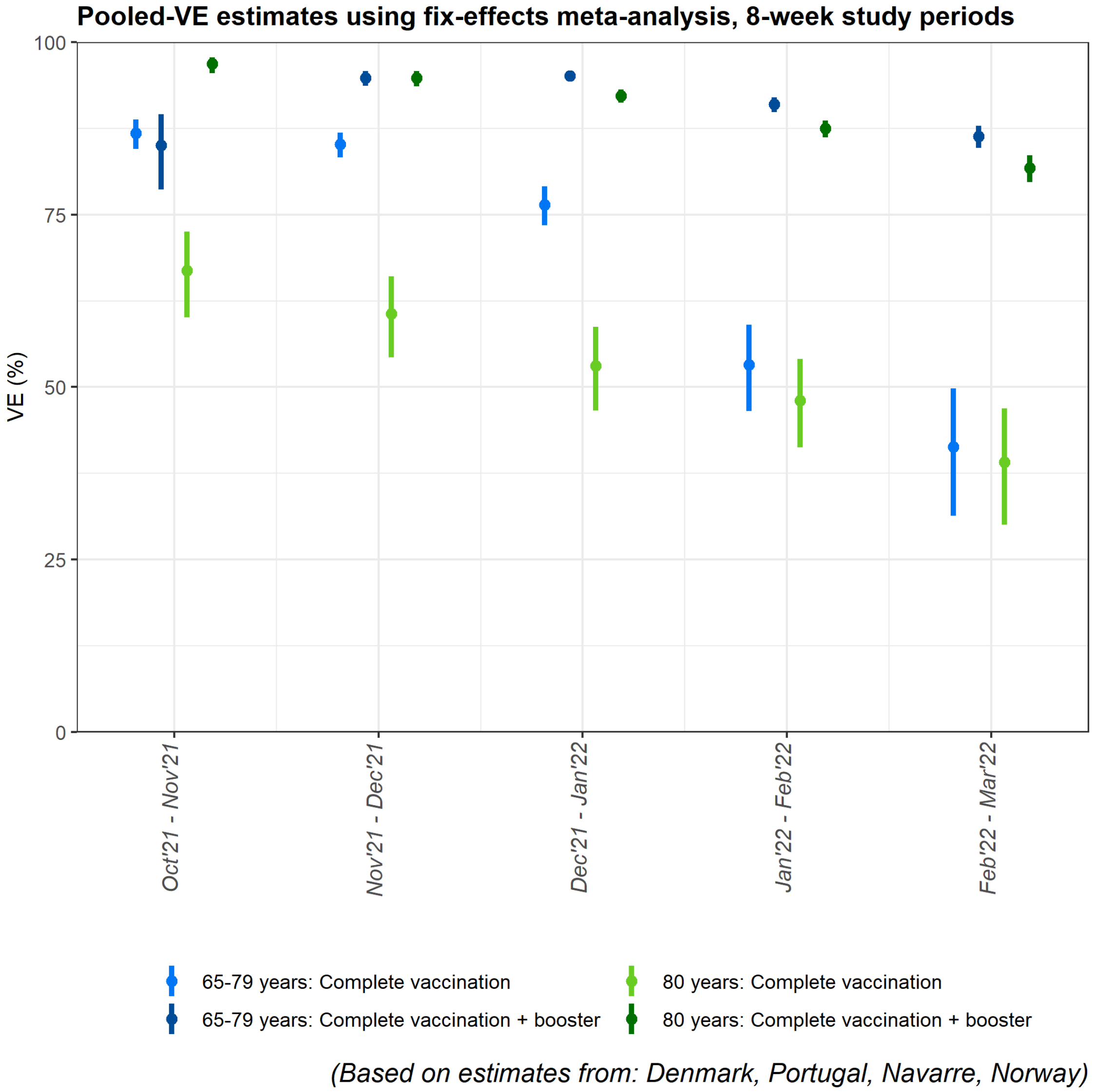

Age	Observation period	VE of complete vaccination	VE of complete vaccination + first booster
**65-79**	October 1 to November 25, 2021	86.8% (84.5, 88.8)	85.0% (78.6, 89.5)
	November 1 to December 26, 2021	85.2% (83.3, 86.9)	94.9% (93.7, 95.8)
	December 1, 2021 to January 25, 2022	76.4% (73.4, 79.1)	95.1% (94.5, 95.7)
	January 1 to February 25, 2022	53.2% (46.5, 59.1)	91.0% (89.9, 92.0)
	February 1 to March 28, 2022	41.3% (31.3, 49.8)	86.4% (84.7, 87.9)
**80+**	October 1 to November 25, 2021	66.9% (60.1, 72.6)	96.8% (95.5, 97.8)
	November 1 to December 26, 2021	60.6% (54.3, 66.1)	94.8% (93.6, 95.8)

	December 1, 2021 to January 25, 2022	53.1% (46.6, 58.7)	92.2% (91.2, 93.2)
	January 1 to February 25, 2022	48.1% (41.3, 54.1)	87.5% (86.2, 88.6)
	February 1 to March 28, 2022	39.1% (30.1, 46.9)	81.8% (79.7, 83.6)

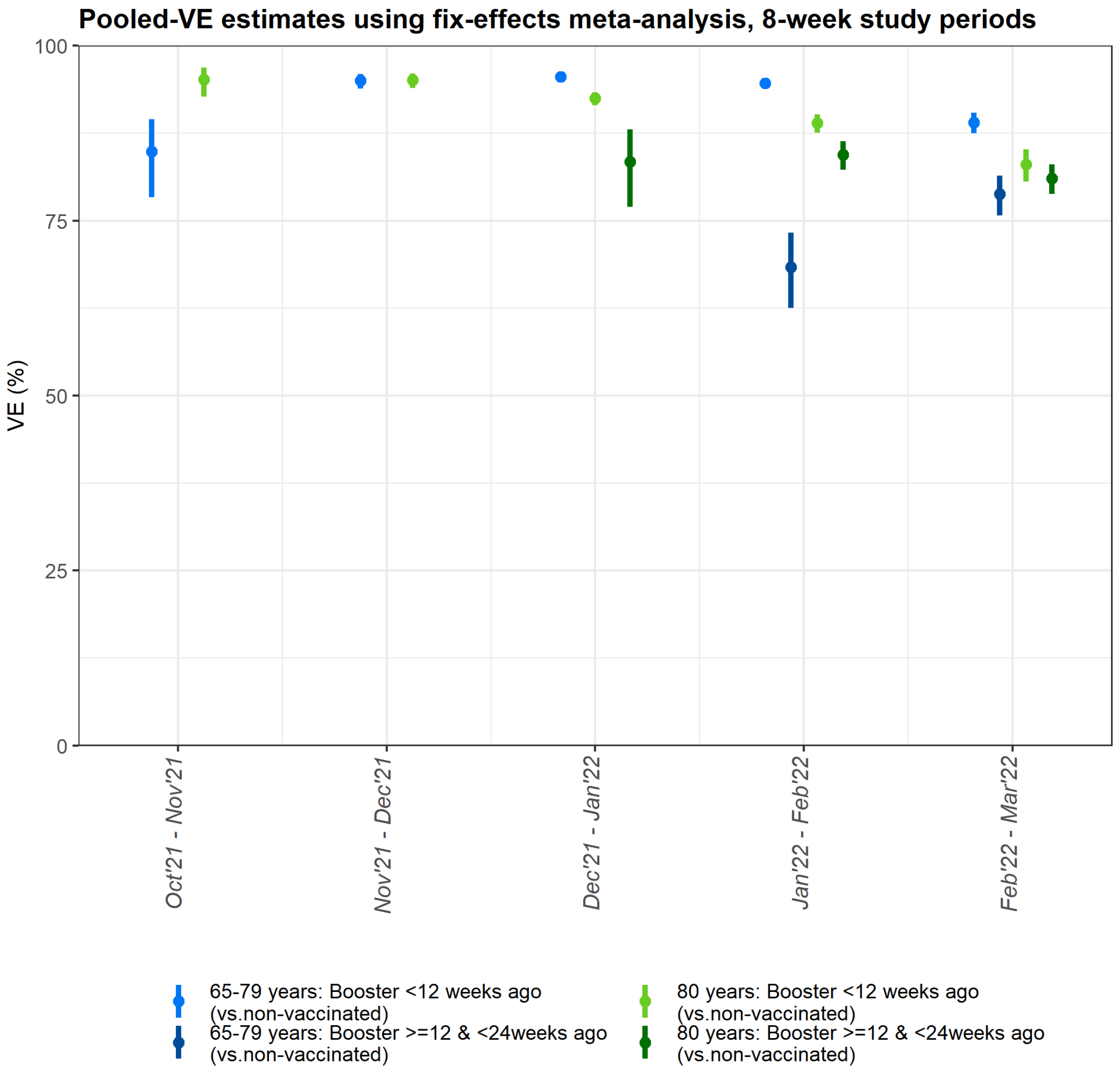

(Based on estimates from: Denmark, Portugal, Navarre, Norway)

Age	Observation period	VE of complete vaccination + first booster < 12 weeks ago	VE of complete vaccination + first booster ≥12 and <24 weeks ago
**65-79**	October 1 to November 25, 2021	84.9% (78.3, 89.5)	NA
	November 1 to December 26, 2021	95.0% (93.9, 95.9)	NA
	December 1, 2021 to January 25, 2022	95.5% (94.9, 96.1)	57.6% (43.7, 68.1)
	January 1 to February 25, 2022	94.6% (94.1, 95.1)	68.3% (62.5, 73.2)
	February 1 to March 28, 2022	89.0% (87.5, 90.4)	78.8% (75.7, 81.4)

**80+**	October 1 to November 25, 2021	95.2% (92.7, 96.8)	NA
	November 1 to December 26, 2021	95.1% (93.9, 96.0)	NA
	December 1, 2021 to January 25, 2022	92.5% (91.5, 93.4)	83.4% (77.0, 88.0)
	January 1 to February 25, 2022	89.0% (87.6, 90.2)	84.4% (82.3, 86.3)
	February 1 to March 28, 2022	83.1% (80.6, 85.2)	81.0% (78.8, 83.0)
